# Hydroxyapatite microspheres induce durable pleurodesis and are rapidly cleared by pleural osteoclasts

**DOI:** 10.1172/jci.insight.192981

**Published:** 2025-08-21

**Authors:** Yusuke Tanaka, Yuki Takahashi, Yuma Shindo, Lori B. Pitstick, Steven L. Teitelbaum, Wei Zou, Xiangning Wang, Jason C. Woods, Kathryn A. Wikenheiser-Brokamp, Francis X. McCormack

**Affiliations:** 1 Division of Pulmonary, Critical Care and Sleep Medicine, Department of Internal Medicine, University of Cincinnati, Cincinnati, Ohio, USA.; 2 Department of Pathology and Immunology, and Division of Bone and Mineral Diseases, Department of Medicine, Washington University School of Medicine, St. Louis, Missouri, USA.; 3 Vontz Core Imaging Laboratory, Vontz Center for Molecular Studies, The University of Cincinnati, Cincinnati, Ohio, USA.; 4 Center for Pulmonary Imaging Research, Cincinnati Children’s Hospital Medical Center, Cincinnati, Ohio, USA.; 5 Division of Pathology & Laboratory Medicine and Perinatal Institute, and; 6 Division of Pulmonary Biology, Cincinnati Children’s Hospital Medical Center, Department of Pathology & Laboratory Medicine, University of Cincinnati, Cincinnati, Ohio, USA.

**Keywords:** Bone biology, Pulmonology, Monocytes, Osteoclast/osteoblast biology, Respiration

## Abstract

Talc pleurodesis is highly effective for preventing recurrence of pneumothorax and pleural effusion, but it can be complicated by dissemination, acute lung injury, lead exposure, and foreign body–induced chronic inflammation and pain. Our objective is to develop a safe, biodegradable, contaminant-free particle for pleurodesis. We used mouse models of pneumothorax and malignant pleural effusion to compare the efficacy and safety of pleurodesis with talc and hydroxyapatite microspheres (HAM). Intrapleural instillation of microspheres induced pleural adhesions, fibrosis, and symphysis as effectively as talc and resulted in more durable protection from experimental pneumothorax. HAM and talc both induced an osteoclastogenic, inflammatory, and fibrotic response in pleural lavage cells. Intrapleural HAM was resorbed by osteoclast action over 3 months, whereas talc was not cleared. Deletion of the osteoclast effector, CTSK, diminished pleural adhesion formation and fibrosis by HAM, and inhibition of osteoclastogenesis with anti-RANKL antibody delayed HAM clearance. We found no difference in activity level, feeding behavior, or lung compliance between particles, but talc induced more persistent pleural inflammation. We conclude that HAM resulted in an osteoclastogenic and fibrogenic pleural response that induced pleurodesis that was more durable than talc with a superior safety profile due in part to osteoclast-mediated particle clearance.

## Abstract 2

Hydroxyapatite microspheres composed of calcium phosphate are a biodegradable alternative to talc for pleurodesis of recurrent pneumothorax (lung collapse) or pleural effusion (fluid collection).

## Graphical abstract

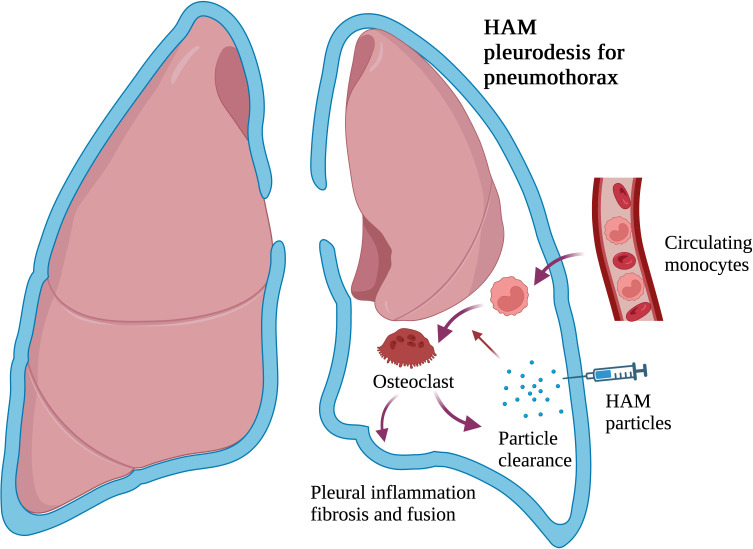

## Introduction

Recurrent or persistent pleural effusions and pneumothoraces are managed by performing pleurodesis, a method to obliterate the potential space between the visceral and parietal pleura by inducing inflammation and fibrotic fusion of the membranes (1, 2). The less invasive methods of pleurodesis include delivery of sclerosants such as doxycycline, bleomycin, or talc slurry into the pleural space via a chest tube or catheter (3), and thoracoscopic surgical options include mechanical abrasion of the parietal pleura with gauze, pleurectomy involving removal of strips of parietal pleura to expose fibrogenic tissue underneath, or talc poudrage to coat the surface of the lung with talc powder under direct visualization (4). Of these approaches, those involving talc are the most effective at inducing pleural fusion and preventing recurrences, but they are also the most fraught because the particles are never cleared, are contaminated with low levels of lead that limit use in children and pregnant women, and can be associated with acute respiratory distress syndrome (ARDS), dissemination, and chronic inflammation and chest pain (5–9). The objective of this work was to develop a biodegradable particle for pleurodesis that retains the efficacy of talc but does not suffer from its limitations and complications.

The inspiration for this research direction came from the study of a rare pulmonary disease, pulmonary alveolar microlithiasis, in which a missing sodium phosphate cotransporter in the lung epithelium results in accumulation of phosphate in the alveolar lining fluid that complexes with calcium and forms bone-like, hydroxyapatite [Ca_5_(PO_4_)_3_(OH)] microliths in the distal airspaces of the lung (10). We found that the lung responds to this particulate challenge by reprogramming the alveolar epithelium and pulmonary lymphocytes to produce RANKL, the primary osteoclastogenic cytokine, to differentiate recruited alveolar monocytes into bone resorbing osteoclasts (11). Intratracheal silica challenge produced a similar osteoclastogenic response, and we found that inhibiting osteoclast differentiation attenuated silica-induced lung remodeling and fibrosis, likely by limiting elaboration of hydrochloric acid and matrix degrading enzymes by lung osteoclasts (12). Finally, we discovered that, after intratracheal challenge with natural or synthetic hydroxyapatite microliths, the particles were resorbed without residual inflammation or fibrosis within a month. Collectively, these observations led us to postulate that intrapleural hydroxyapatite would result in a similar program of recruitment and differentiation of circulating monocytes into osteoclasts, secretion, or release of acid and enzymes that drive collateral tissue injury leading to fibrotic fusion of pleural membranes and clearance of the particle over time. A successful outcome of these preclinical studies could set the stage for pleurodesis trials with a biodegradable particle that is as effective as talc but that has a more favorable safety profile.

## Results

**Figure 1:**
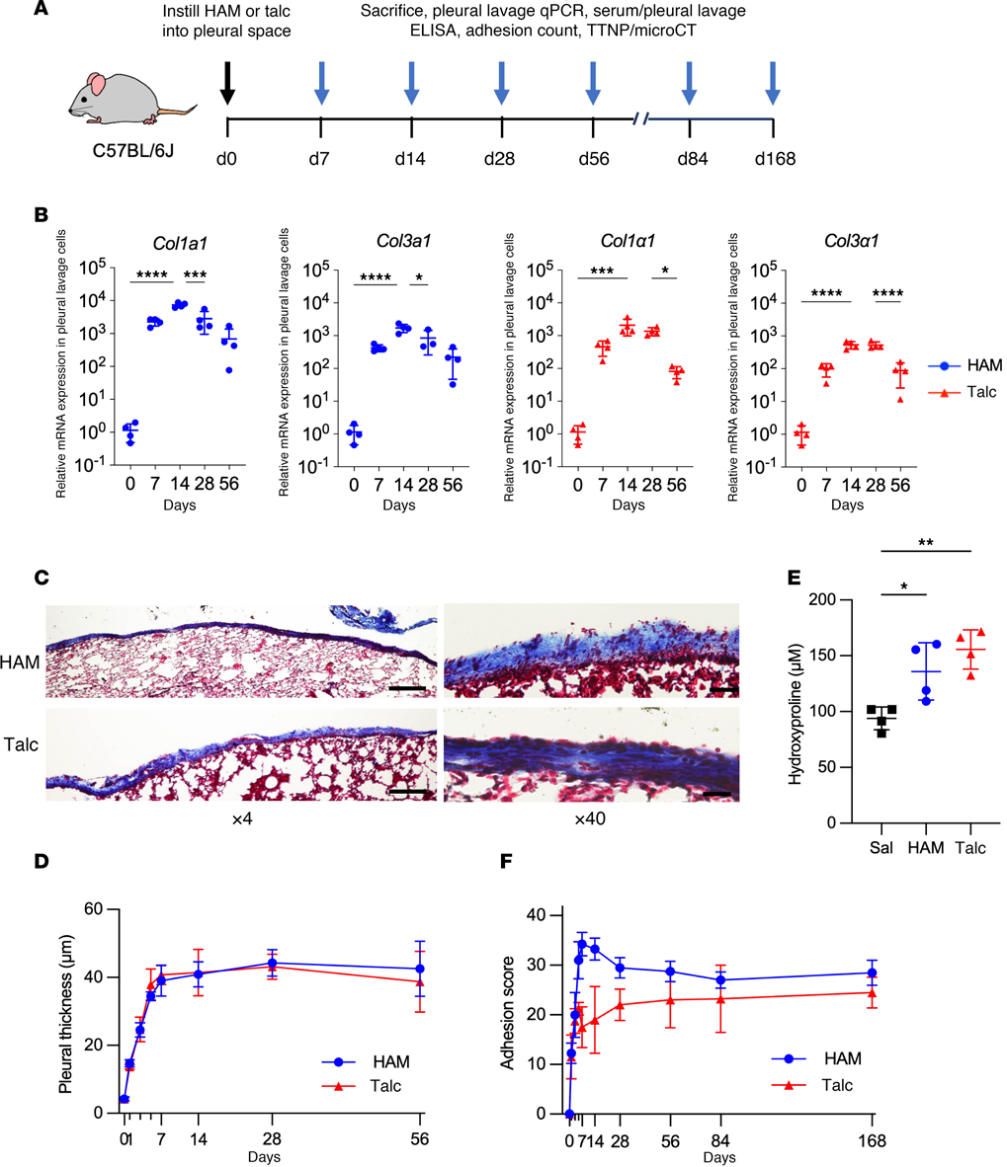
Comparison of HAM and talc induced pleural fibrosis and fusion. (**A**) Scheme for pleurodesis experiments with HAM and talc. (**B**) qPCR of collagen genes, *Col1a1* and *Col3a1*, from pleural lavage cells at indicated intervals after HAM and talc administration (*n* = 4 mice per group). (**C**) Masson’s trichrome reagent–stained lung sections collected from HAM and talc challenged mice at 56 days. Scale bar: 200 μm for ×4 magnification and 20 μm for ×40 magnification. (Supplemental Figure 2A). (**D**) Pleural thickness change after intrapleural administration of HAM and talc (Supplemental Figure 2B) (*n* = 4 mice per group). (**E**) Lung surface and whole lung (Supplemental Figure 2C) hydroxyproline levels were quantified at day 14 after intrapleural administration of HAM or talc (*n* = 4–8 mice per group). (**F**) Adhesions between the visceral and parietal pleura were quantified at defined time points after the intrapleural administration of HAM or talc (Supplemental Figure 2D). Data are shown as mean ± SD. Comparisons were by unpaired *t* test for 2 groups and by 1-way ANOVA followed by Tukey’s multiple-comparison test. *P < 0.05, **P < 0.01, ***P < 0.001, and ****P < 0.0001.

We compared the efficacy of laboratory talc and commercially available hydroxyapatite microspheres (HAM) for pleurodesis in mice. The particles used were of similar mean sizes (8.2 ± 2.2 μm vs. 6.0 ± 2.7 μm, mean ± SD for talc vs. HAM, respectively) (Supplemental Table 1; supplemental material available online with this article; https://doi.org/10.1172/jci.insight.192981DS1), but the physical characteristics were quite distinct, with talc exhibiting a rough, irregular, plate-like shape with a fractured, layered surface and with HAM showing a near perfectly spherical shape with a smooth, textured, porous surface (Supplemental Figure 1). Talc and HAM doses that were delivered (2 mg per gram body weight [BW] vs. 4 mg/g BW) were based on the maximal recommended dose for talc in humans (10 g) corrected for body surface area (1.73 m^2^ for humans and 0.007 m^2^ for mice), and designed to compensate for the greater surface area of talc (7–25 m^2^/g) vs. HAM (0.2–0.4 m^2^/g) based on weight, and greater formula weight of HAM (1,004 g/mol) compared with talc (347 g/mol), which results in fewer particles delivered per unit weight (Supplemental Table 2). The particles were instilled into the pleural space, and pleural fibrosis was assessed by several measures at multiple time points (Figure 1A). Upregulation of collagen genes in pleural lavage cells was induced by both intrapleural HAM and talc, peaking at day 14 and dropping toward baseline through day 56 (Figure 1B). Pleural thickness and expansion of the collagen matrix was measured on Masson’s trichrome stained sections, and they were found to increase sharply after intrapleural instillation over 7 days and to remain elevated through day 56 to a very similar degree for both particles (Figure 1, C and D, and Supplemental Figure 2, A and B). The hydroxyproline content of the lung surface (Figure 1E) and whole lung (Supplemental Figure 2C) was also increased to a similar extent for talc and HAM. Pleural adhesion scores in mice treated with intrapleural particles were higher in the HAM group in the first 2 months after intrapleural but converged with those due to talc by day 84 and day 168 after challenge (Figure 1F and Supplemental Figure 2D). The static compliance of the lung was decreased at day 56 to a similar degree for both particles (Supplemental Figure 2E), consistent with the development of a mild restrictive defect.

**Figure 2:**
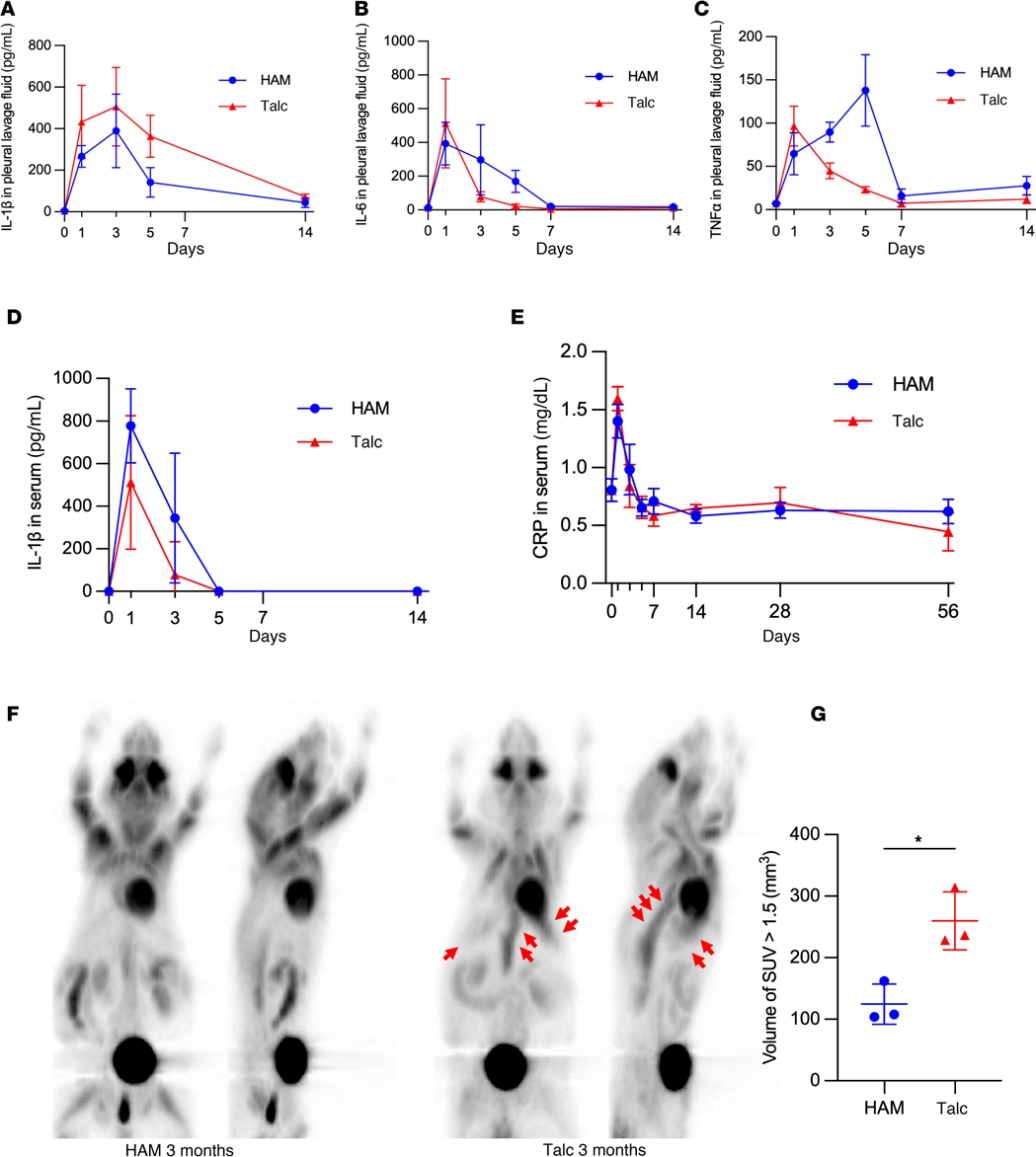
Pleural and systemic inflammation associated with HAM and talc pleurodesis. (**A**–**C**) Analysis of IL-1β, IL-6, and TNF-α in pleural lavage fluid measured by ELISA at indicated time points after administration of HAM or talc (*n* = 4 mice per group) (Supplemental Figure 3, A–C). (**D** and **E**) IL-1β and CRP in serum measured by ELISA at indicated time points after administration of HAM or talc (*n* = 4 mice per group) (Supplemental Figure 3, D and E). (**F**) Residual inflammation on FDG-PET at 3 months after administration of HAM or talc (red arrows). (**G**) The volume voxels with standardized uptake value (SUV) > 1.5 is shown (*n* = 3). Data are shown as mean ± SD. *P < 0.05.

We compared the inflammatory responses induced by HAM and talc pleurodesis. Both HAM and talc induced an increase in IL-1β, IL-6, and TNF-α in pleural lavage fluid that peaked over the first week and returned to baseline for the next 7–14 days after instillation (Figure 2, A–C, and Supplemental Figure 3, A–C). Serum IL-1β and the acute phase reactant C-reactive protein (CRP) followed a similar pattern for both particles (Figure 2, D and E, and Supplemental Figure 3, D and E). Residual ¹^8^F-fluorodeoxyglucose (FDG) uptake in the thoracic cavity on PET scanning at 3 months after talc instillation was significantly greater than that for HAM, based on comparison of volume of voxels with standardized uptake value (SUV) > 1.5, consistent with greater ongoing inflammation in the pleural space with the nondegradable particle (Figure 2, F and G).

**Figure 3:**
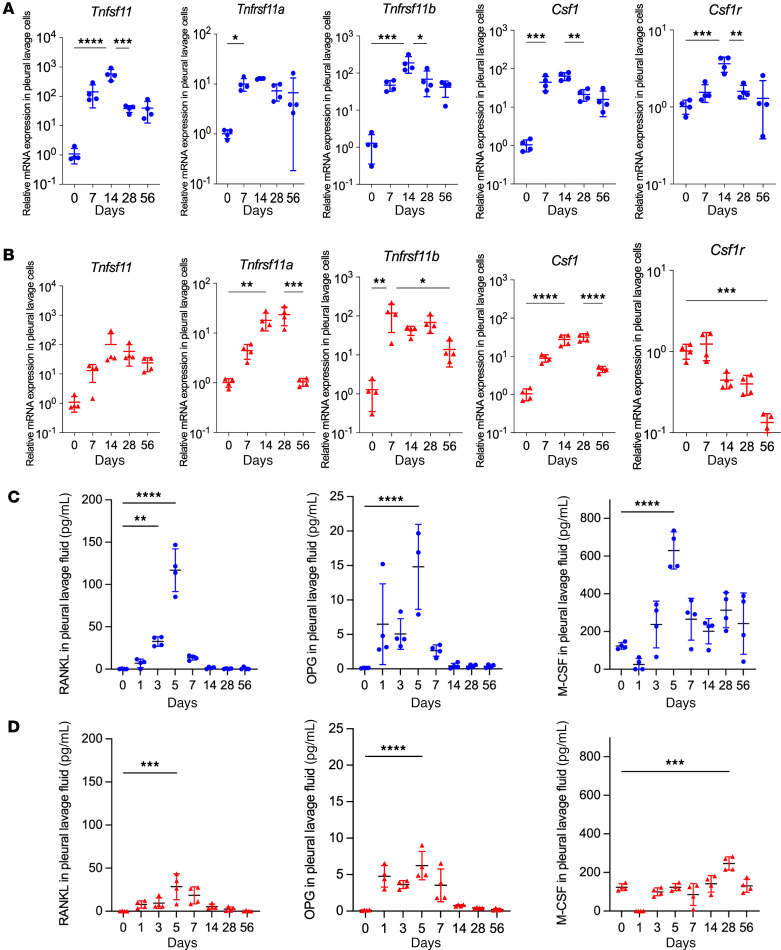
Osteoclastogenic cytokines induced by HAM and talc pleurodesis. (**A** and **B**) qPCR for osteoclastogenic genes *Tnfsf11* (RANKL), *Tnfrsf11a* (RANK), *Tnfrsf11b* (OPG), *Csf1* (M-CSF), and *Csf1r* (M-CSFR), in pleural lavage cells collected at indicated time points after intrapleural instillation of HAM (blue) (**A**) or talc (red) (**B**) (*n* = 4 mice per group). ELISA of RANKL, OPG, and M-CSF proteins in pleural lavage fluid over time after instillation of HAM (blue) (**C**) or talc (red) (**D**). Data are shown as mean ± SD. Comparisons were by unpaired *t* test for 2 groups and by 1-way ANOVA followed by Tukey’s multiple-comparison test. *P < 0.05, **P < 0.01, ***P < 0.001, and ****P < 0.0001.

We explored the hypothesis that intrapleural particles induce an osteoclastogenic gene program that both contributes to clearance (as we have shown for hydroxyapatite; refs. 10, 11) and results in release of damaging cellular products that promote fibrosis (as we have shown for silica; ref. 12) and pleural symphysis. Osteoclastogenic cytokine genes, *Tnfsf11* (RANKL), *Tnfrsf11a* (RANK), *Tnfrsf11b* (OPG), *Csf1* (M-CSF), and *Csf1r* (M-CSFR) were induced in pleural lavage cells by day 7 for both HAM and talc, plateaued on day 14–28, and remained elevated above baseline through day 56 (Figure 3, A and B). Pleural lavage fluid levels of RANKL, OPG, and M-CSF proteins were elevated to a greater degree (3- to 5-fold) by HAM (Figure 3C) than by talc (Figure 3D), peaking at day 5 and falling to baseline in the second week after instillation.

**Figure 4:**
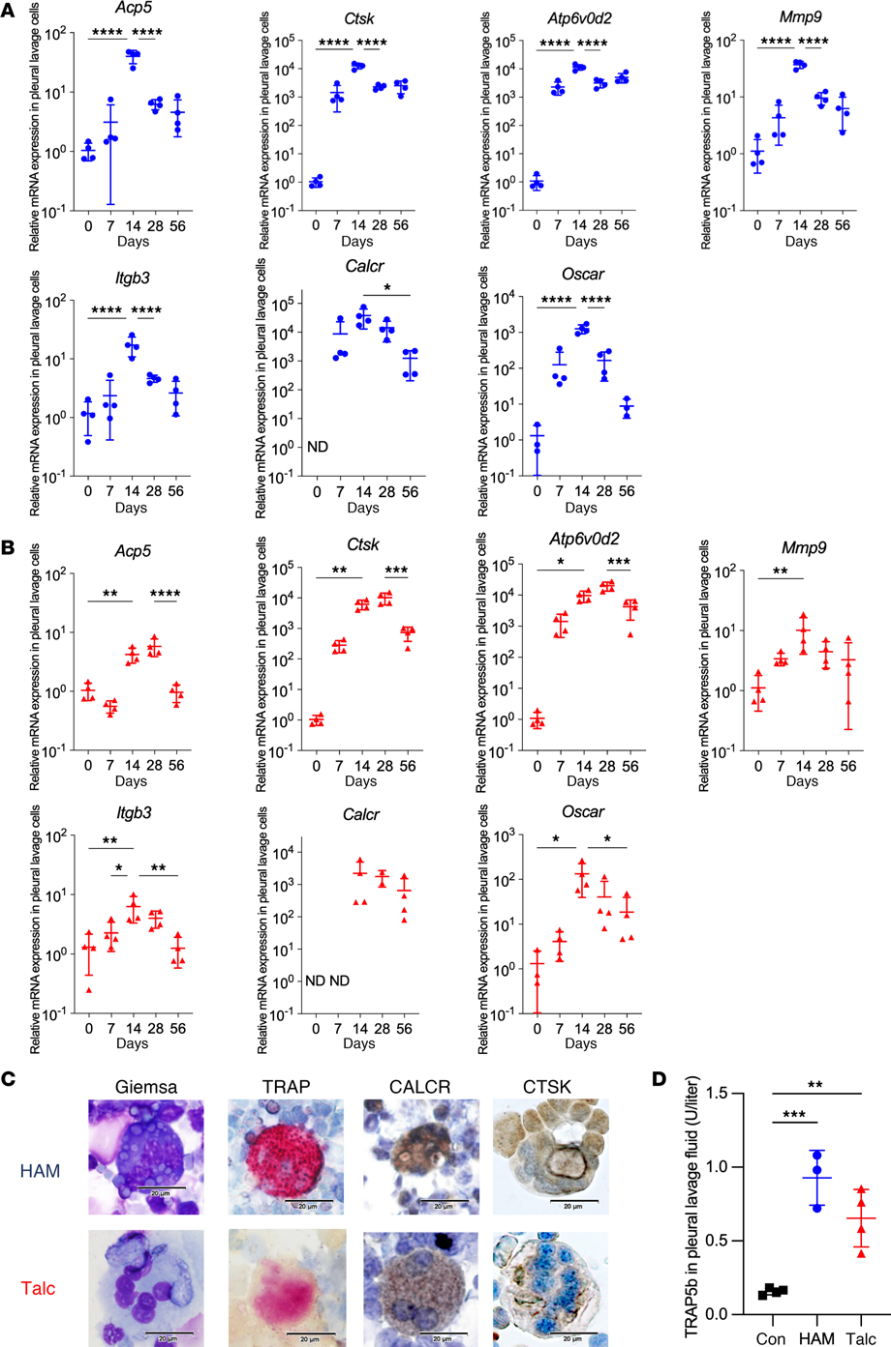
Osteoclast genes and proteins induced by HAM and talc pleurodesis. (**A** and **B**) qPCR for osteoclast genes *Acp5* (TRAP), *Ctsk*, *Atp6v0d2*, *Mmp9*, *Itgb3* (Integrin β3) and *Calcr* (Calcitonin receptor), and *Oscar* (Osteoclast-associated immunoglobulin-like receptor) in pleural lavage cells collected at the indicated time points after intrapleural instillation of HAM (blue) (**A**) or talc (red) (**B**). (**C**) Pleural multinucleated giant cells at 14 days after intrapleural challenge of HAM or talc stained with Giemsa, or for TRAP, CALCR, and CTSK. Scale bars: 20 μm. (**D**) TRAP5b, quantified by ELISA, in pleural lavage fluid from control, HAM- or talc-treated mice collected at 14 days after challenge (*n* = 3–4 mice per group). Data are shown as mean ± SD. Comparisons were by unpaired *t* test for 2 groups and by 1-way ANOVA followed by Tukey’s multiple-comparison test. *P < 0.05, **P < 0.01, ***P < 0.001, and ****P < 0.0001.

As shown in Figure 4, A and B, HAM and talc induced a 1–4 log upregulation of multiple osteoclast effector genes in pleural lavage cells that peaked at day 14 after challenge and remained elevated through day 56, including tartrate resistant acid phosphatase (TRAP; *Acp5*), cathepsin K (*Ctsk*), ATPase, H+ transporting, lysosomal 38 kDa, V0 subunit d2 (*Atp6v0d2*), matrix metalloproteinase 9 (*Mmp9*), calcitonin receptor (*Calcr*), and osteoclast-associated immunoglobulin-like receptor (*Oscar*). Both HAM and talc induced formation of multinucleated TRAP^+^, CALCR^+^, and CTSK^+^ giant cells in pleural lavage fluid (Figure 4C), as well as an increase in pleural lavage fluid levels of the osteoclast specific isoform of TRAP, TRAP5b (Figure 4D).

**Figure 5:**
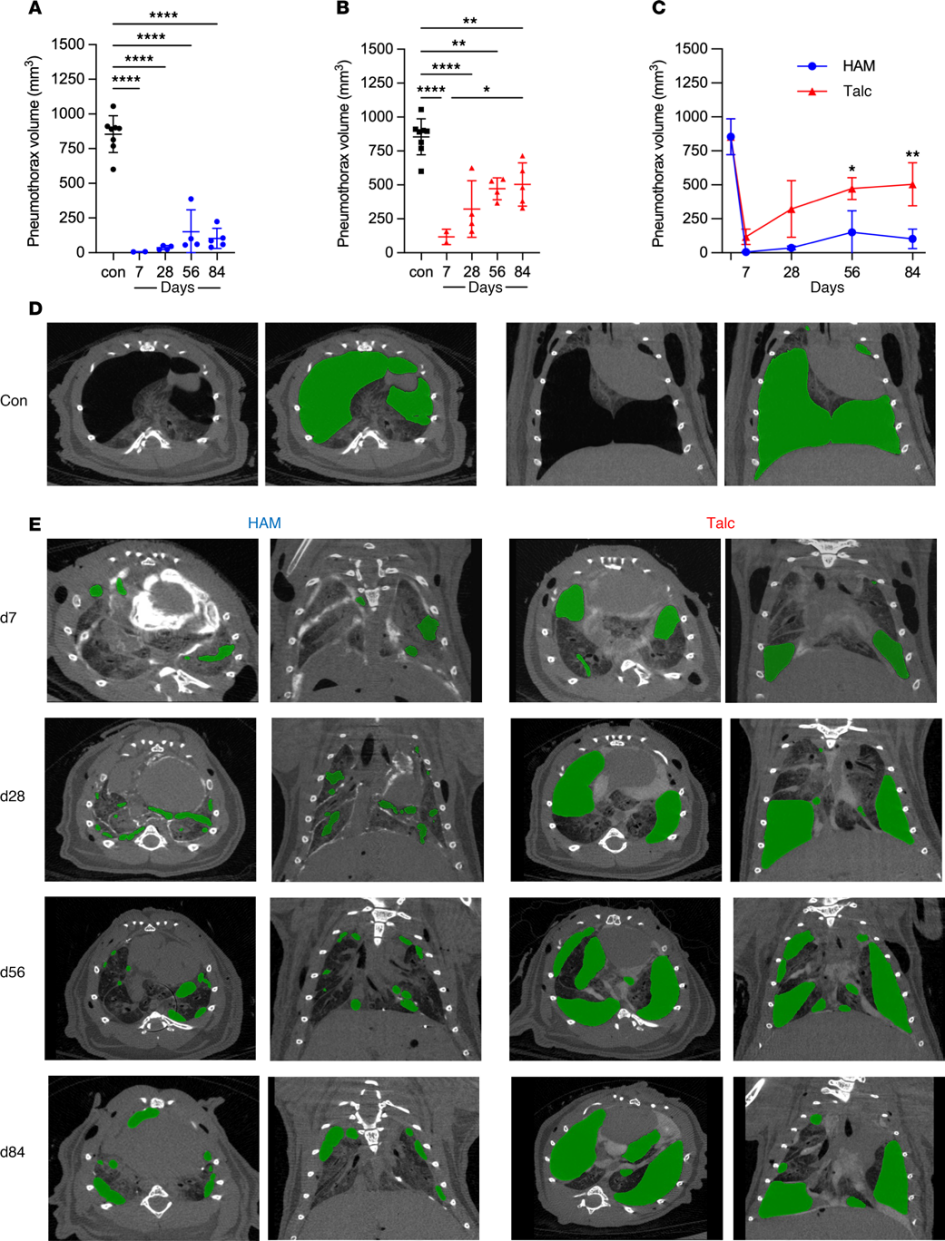
Effectiveness of HAM and talc for pleurodesis. Mice were sacrificed at the indicated time points after intrapleural instillation of particles and transthoracic needle puncture (TTNP) was performed with multiple passes of a 20 gauge needle through both hemithoraces. (**A**–**C**) μCT was used to quantify the volume of pneumothorax for HAM (**A**) and talc (**B**), which were compared head to head in **C**. (**D**) For control, axial and coronal CT images of saline challenged mice after TTNP are shown, as well as companion images colorized to highlight air in the pleural space. (**E**) For HAM and talc treated mice, colorized axial and coronal CT images are shown, as well as corresponding unadulterated images (Supplemental Figures 4A [HAM] and 4B [talc]). Data are shown as mean ± SD. Comparisons were by unpaired *t* test for 2 groups and by 1-way ANOVA followed by Tukey’s multiple-comparison test. *P < 0.05, **P < 0.01, ***P < 0.001, and ****P < 0.0001.

Postmortem transthoracic needle puncture (TTNP) is a method developed for inducing pneumothoraces in freshly sacrificed animals at defined time points after intrapleural instillation of saline or particles by multiple passes of a 20-gauge needle through each hemithorax. Quantitative μCT is used to estimate the volume of pneumothorax before and after intrapleural administration of particles to evaluate the effectiveness of pleurodesis (Figure 5). In control animals given intrapleural saline, TTNP induced large pneumothoraces with volumes of greater than 900 mm^3^ (Figure 5). Following intrapleural instillation of either HAM or talc, residual pneumothorax volume was reduced to less than 100 mm^3^ at the 1-week time point (Figure 5, A–C). Over the course of the ensuing 12 weeks, HAM pleurodesis was generally more durable than that due to talc, such that by the 12-week point, pneumothorax volume was approximately two-thirds of control for talc and one-eighth of control for HAM (Figure 5, A–C). Colorized CT images taken at the day 7, 28, 56, and 84 time points after particle instillation highlight the greater reduction in pneumothorax volume by HAM compared with talc (Figure 5, D and E, and Supplemental Figure 4, A and B). Thus, effectiveness of HAM pleurodesis appeared to be superior to talc pleurodesis in the mouse model over a 3-month period.

**Figure 6:**
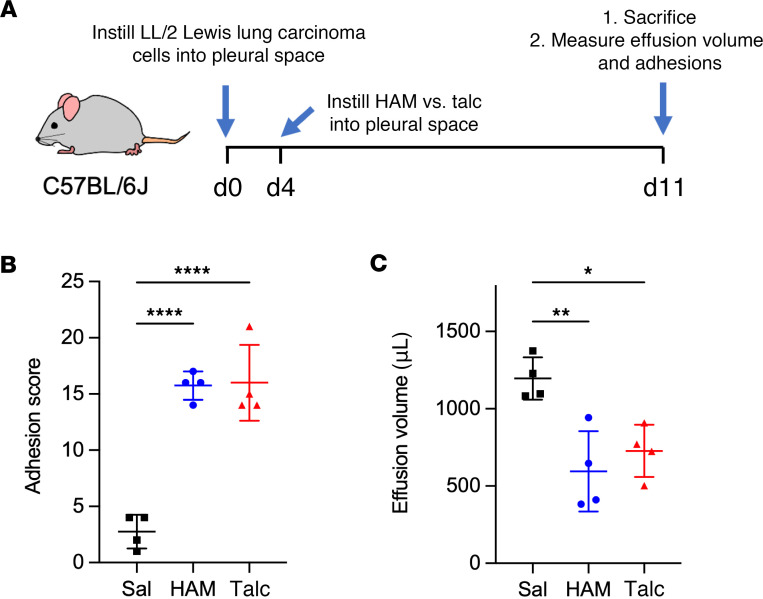
Effectiveness of HAM and talc pleurodesis for malignant effusion. (**A**) Mice were treated with intrapleural HAM or talc 4 days after intrapleural instillation of 5 × 10^4^ Lewis lung carcinoma (LL/2) cells into the pleural space. (**B** and **C**) One week later, the animals were sacrificed and number of pleural adhesions (**B**) and volume of pleural effusion (**C**) were quantified. Data are shown as mean ± SD. Comparisons were by unpaired *t* test for 2 groups and by 1-way ANOVA followed by Tukey’s multiple-comparison test. *P < 0.05, **P < 0.01, and ****P < 0.0001.

Talc pleurodesis is commonly used to treat malignant pleural effusions. We tested this application of hydroxyapatite pleurodesis in a mouse model that employs intrapleural instillation of Lewis lung carcinoma (LL/2) cells (13) (Figure 6A) to establish a malignant pleural effusion. Four days after LL/2 cells were delivered to the pleural space, we instilled HAM or talc, and we then measured the adhesion scores (Figure 6B) and effusion volumes (Figure 6C) over time. We found that both HAM and talc performed similarly, with nearly identical adhesion scores and an approximate 50% reduction in malignant pleural fluid volume. BW fell to a similar degree after particle instillation (Supplemental Figure 5). These data suggest that HAM is as effective as talc for pleurodesis in mice with malignant pleural effusion.

**Figure 7:**
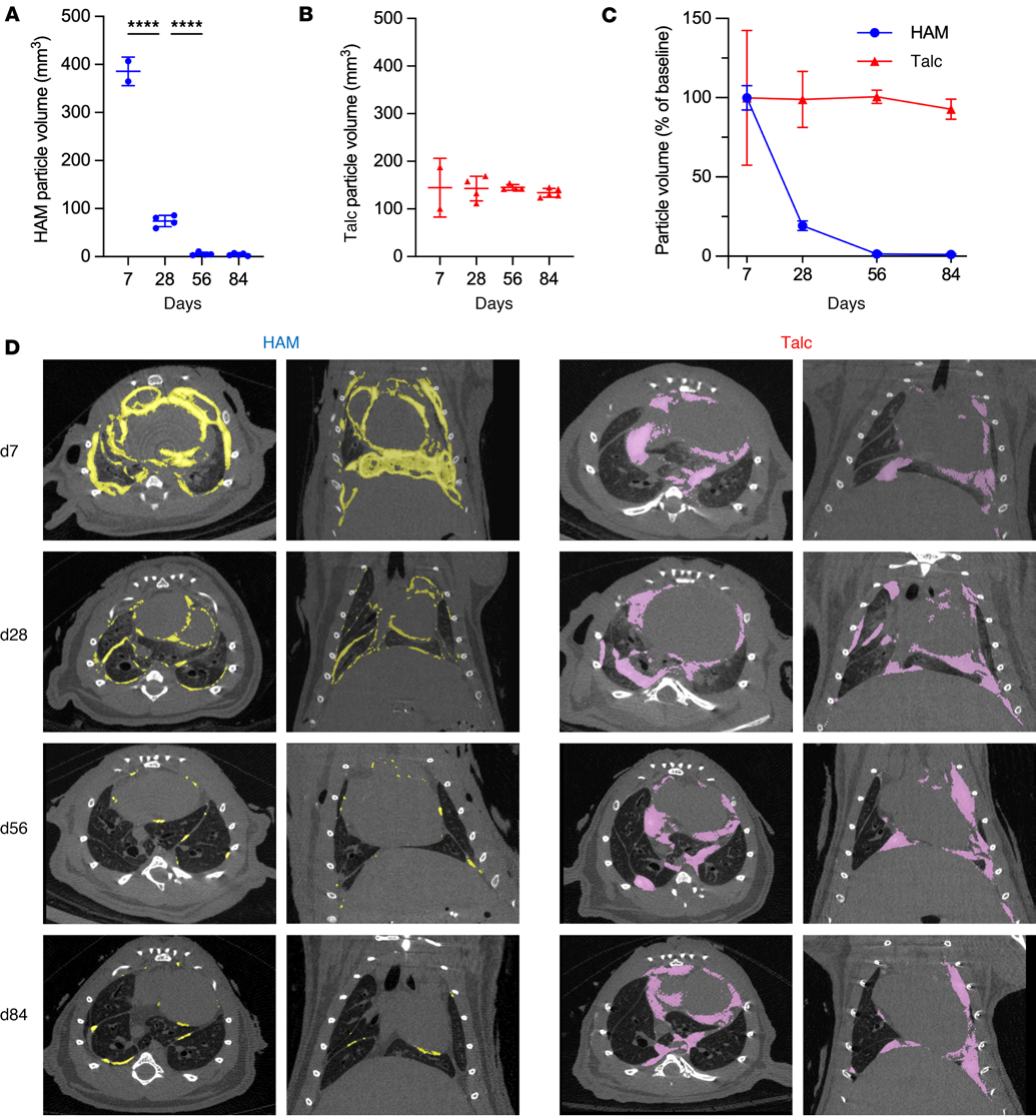
Clearance of HAM and talc particles over time. Mice that had been treated with intrapleural HAM or talc were anesthetized and imaged with μCT, and the volume of voxels attributable to particles was estimated using quantitative methods. (**A**–**C**) The particle voxel volume for HAM (**A**) and talc (**B**) are shown, and compared head to head in **C**. (**D**) Axial and coronal CT colorized images are shown, as well as unadulterated CT images for HAM- (Supplemental Figure 6A) and talc-treated (Supplemental Figure 6B) animals. Data are shown as mean ± SD. Comparisons were by unpaired *t* test for 2 groups and by 1-way ANOVA followed by Tukey’s multiple-comparison test. ****P < 0.0001.

We also compared the clearance of radio-opaque intrapleural HAM or talc particles by quantitative μCT over the course of 3 months (Figure 7 and Supplemental Figure 6). Colorization of CT images was used to highlight residual particles. HAM particles were widely distributed and profuse at day 7 and progressively cleared through day 28, reaching nearly undetectable levels by day 56, while talc particle volumes remained unchanged through day 84.

**Figure 8:**
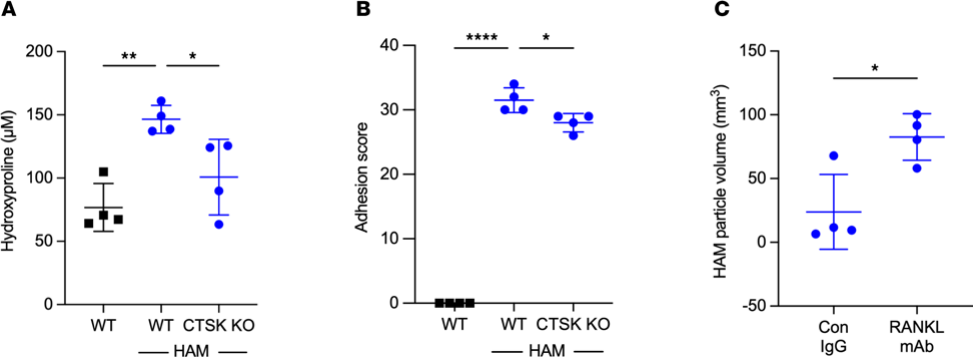
Effects of blocking osteoclast activity on pleural fibrosis and particle clearance. (**A** and **B**) *Ctsk*^–/–^ mice and control littermates were treated with intrapleural HAM and sacrificed on day 14, and pleural surface hydroxyproline (**A**) and adhesion scores (**B**) were determined as in Figure 1. (**C**) In separate experiments, mice were treated with intrapleural HAM and treated with control IgG or anti-RANKL mAb 3 times weekly over 28 days. μCT was performed, and HAM voxel density was estimated by quantitative methods as outlined in Figure 7. Data are shown as mean ± SD. Comparisons were by unpaired *t* test for 2 groups and by 1-way ANOVA followed by Tukey’s multiple-comparison test. *P < 0.05, **P < 0.01, and ***P < 0.001

To determine if osteoclast products may be contributing to pleural symphysis, we measured the adhesion scores in particle-challenged animals that were deficient in matrix degrading enzyme production (*Ctsk*^–/–^ mice) (Figure 8, A and B). We found that there was a significant decline in pleural surface hydroxyproline and pleural adhesion formation at 14 days after HAM challenge in *Ctsk*^–/–^ mice. We did not find any effect of deletion of CTSK on the formation of adhesions due to talc (not shown). In addition, we found that HAM clearance was delayed in mice treated with anti-RANKL mAb 3 days per week for 4 weeks (Figure 8C). These data suggest that induction of osteoclast activity plays a role in adhesion formation as well as in particle clearance in animals treated with intrapleural HAM.

Several studies were conducted to determine the systemic effects and toxicities of HAM and talc pleurodesis (Supplemental Figure 7). Intrapleural administration of both particles induced a small, transient increase in serum calcium levels to a similar degree that returned to baseline by 4 weeks after challenge (Supplemental Figure 7A), but there was no effect of HAM or talc on serum phosphate levels (Supplemental Figure 7B). BW declined initially after intrapleural particle instillation, but it then recovered in both HAM and talc challenged mice over time (Supplemental Figure 8). Talc limited natural BW gain over time to a greater extent than HAM, although the effect size was small and did not reach significance by multiple-comparison testing (Supplemental Figure 8A). In a separate cohort of mice, weights, total activity levels, and total food intake were assessed and were not different between the intrapleural HAM, talc, and saline control groups at day 42 (Supplemental Figure 8B). Collectively, these data suggest that HAM is at least as safe as talc in the mouse model.

**Figure 9:**
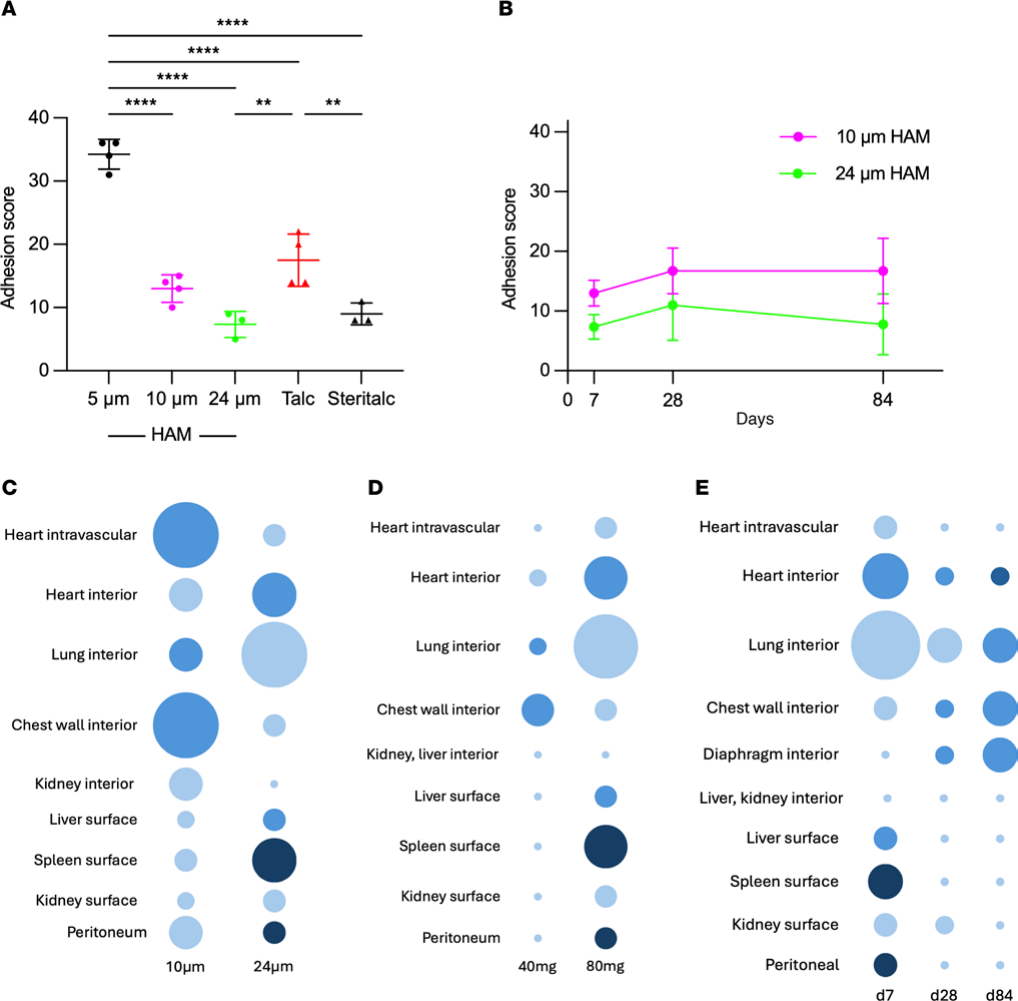
Adhesion formation, dissemination, and clearance of HAM particles. (**A**) Pleural adhesion formation was assessed on day 7 after intrapleural instillation of 80 mg of 5 μm HAM, 10 μm HAM, or 24 μm HAM particles or 40 mg laboratory grade or clinical grade particles as outlined in Methods. (**B**) Intrapleural delivery of 80 mg of 10 μm (pink) or 24 μm (green) HAM particles to mice at time zero was followed by determination of pleural adhesion scores at 7, 28, and 84 days. (**C**–**E**). The effects of particle size (**C**), particle dose (**D**), and time (**E**) on dissemination and clearance are depicted in dot plots that summarize the data shown in Supplemental Figures 9–11. The size of dots relates to the number of animals of the 3–4 mice surveyed per point that had any particles found, and the intensity of color relates to the maximal profusion of particles found in any of the positive animals in the group (on a scale of 1–4+). Data are shown as mean ± SD. **P < 0.01, ***P < 0.001, and ****P < 0.0001.

Finally, we conducted experiments to determine the influence of time and HAM particle size and dose on BW, efficacy of pleurodesis, and degree of dissemination (Figure 9 and Supplemental Figures 9–11). Smaller particles and higher doses of HAM were associated with greater transient weight loss than larger particles and lower doses of HAM, but weights all converged by day 7 (Supplemental Figure 9A and Supplemental Figure 10A). We sized particles using scanning electron microscopy (SEM) (Supplemental Table 1) and compared the properties of the 5 μm (6 μm by SEM) HAM particles and 8.2 μm laboratory talc that were used for all studies to this point, with size fractionated 10 μm (13.6 μm by SEM) and 24 μm (24.8 μm) HAM particles, and 37.1 μm clinical grade talc. There was an inverse relationship between HAM particle size and the degree of adhesion formation by 5, 10, and 24 μm HAM particles at 7 days after intrapleural instillation with the smaller particles demonstrating greater efficacy than larger particles (Figure 9A). Notably, all HAM particles were at least as effective for adhesion formation as clinical grade talc (Steritalc). Although smaller particles induced more adhesion formation than larger particles, it is unlikely that particles < 10 μm will be acceptable for human use based on available safety data from rodent models (14) and clinical studies suggesting an inferior safety profile (7, 8, 11, 15–18). We therefore focused on better characterizing the safety and efficacy of the 10 and 24 μm HAM particles. Both the 10 and 24 μm HAM particles induced durable pleurodesis, based on stability of adhesion scores through days 28 and 84 (Figure 9B). To determine size dependence of dissemination, we delivered 80 mg of either 10 or 24 μm HAM to the pleural space, which — on a mg per m^2^ basis — is approximately twice the maximal (10 g) recommended dose for Steritalc in humans (Supplemental Table 2). Tissues were harvested 1 week later and von Kossa stained to highlight particles (Figure 9, C–E and Supplemental Figure 9, B–D). In the dot blots shown in Figure 9, C–E, the size of dots relates to the number of animals of the 3–4 mice surveyed per point that had any particles found, and the intensity of color relates to the maximal profusion of particles found in any of the positive animals in the group (on a scale of 1–4+). We found that particles of both sizes were abundant on pleural surfaces of the thorax and studded the peritoneal surfaces of the liver, kidney, and spleen, occurring in 1/4 to 2/3 of animals without a clear size dependence (Figure 9C and Supplemental Figure 9, B–D). These particles almost certainly gained access to the abdominal structures through hiatal openings, lymphatic channels, or stomata in the diaphragm, rather than through intravascular dissemination. The smaller particles were more likely to enter the chest wall, found there in 4/4 animals given 10 μm HAM and 1/3 animals given the 24 μm HAM. In addition, intravascular particles indicative of true dissemination were found within the heart in 4/4 animals given 10 μm particles and 1/3 animals given the 24 μm particles (Figure 9C and Supplemental Figure 9B). Both sized particles were found in the interior tissues of the heart, in 2/4 of the mice in 10 μm group and 2/3 of the mice in the 24 μm group (Figure 9C and Supplemental Figure 9B). von Kossa staining of the heart valve is difficult to interpret, since melanin present in those structures also stain with that reagent (19). Finally particles in the interior tissues of the kidney were found in 2/4 of animals given the 10 μm particles and none of animals given the 24 μm particles. No particles were found in the brain (data not shown). These data suggest that smaller particles (with higher surface area on a mg-for-mg basis) produce more pleural adhesions, but that they also induce more weight loss and are more prone to disseminate intravascularly and into the chest wall, most likely through lymphatic stomata on the parietal pleural surface.

Additional experiments were conducted to determine the dose dependence of adhesion formation and dissemination by the larger (24 μm) particle over a 7-day period (Figure 9D and Supplemental Figure 10, B and C). There was a direct relationship between 24 μm HAM dose (10 mg, 40 mg and 80 mg) and extent of adhesion formation (Supplemental Figure 10B). There was more peritoneal studding, intravascular dissemination, and heart wall invasion for animals given the highest HAM dose, but for unclear reasons, chest wall invasion was seen more often in 40 mg than the 80 mg group (2/4 vs. 1/3, respectively) (Figure 9D and Supplemental Figure 10C). These data suggest that there is a direct relationship between intrapleural HAM dose, weight loss, pleural adhesion formation, and dissemination.

Experiments were performed to assess the time dependence of particle clearance from pleural, peritoneal, intravascular, and interparenchymal compartments (Figure 9E and Supplemental Figure 11). Over the 3 time points examined at 7, 28, and 84 days after intrapleural instillation of 80 mg of 24 µm HAM, there was a reduction in particle scores for lung interior (3/3, 2/4, 2/4, respectively), heart interior (2/4, 1/4, 1/4, respectively), and intravascular space in the heart (1/3, 0/3, 0/3, respectively), but inconsistent temporal patterns were found in the interior of the chest wall (1/3, 1/4, 2/4) and diaphragm (0/3, 1/4, 2/4) (Supplemental Figure 11A). Compared with the abundant peritoneal studding present on day 7, partial clearance from that membrane was apparent by day 28 (only 1 kidney section positive), and complete clearance had occurred by day 84. Taken together, these data suggest that HAM particles that disseminate and migrate through the diaphragm to stud the peritoneum are cleared over time.

A histological survey was conducted on > 100 H&E and Masson’s trichrome sections of the hearts, lungs, livers, kidneys, and spleens of animals treated with intrapleural HAM, including from mice sacrificed on day 7, 28, and 84 after high dose 24 μm HAM, day 7 after low dose 24 μm HAM, and day 7 after high dose 10 μm HAM (not shown). The degree of inflammation and fibrosis in the pleural space was directly correlated with the burden of particles, but there was no evidence of significant inflammation or fibrosis in other random tissues sections surveyed.

## Discussion

Pleurodesis is defined as the fibrotic fusion of the visceral and parietal pleura to prevent the accumulation of either air or liquid in the pleural space. Pleural fluid evacuation followed by pleurodesis is indicated in symptomatic patients with malignant pleural effusion, recurrent benign effusions, and nonresolving or recurrent pneumothorax (20). The administration of intrapleural talc by insufflation or slurry has long been considered the most effective pleurodesis method but toxicities have limited widespread use as the first line agent for all but malignant pleural effusions as a palliative measure. Development of a pleurodesis approach using a biodegradable particle that retains the fusogenic properties of talc without the complications of acute lung injury, chronic pain, lead contamination, and excessive bleeding at subsequent thoracotomy would be a significant advance for pregnant women, children, and patients with recurrent or problematic pneumothoraces or effusions.

Effective pleurodesis has been reported with a wide variety of agents, including tetracycline, bleomycin, and iodine, and although the mechanisms of pleural symphysis are not completely understood, the induction of inflammation is a common theme (1, 21). Mesothelial cells activated by pleurodesis agents secrete chemokines such as IL-8 and monocyte chemoattractant protein (MCP-1), proinflammatory cytokines IL-1β, TNF-α and IL-6, as well as growth factors, vascular endothelial growth factor (VEGF), platelet-derived growth factor (PDGF), basic fibroblast growth factor (bFGF), and TGF-β (22, 23). Monocytes are recruited from the circulation and contribute to the inflammatory response. The coagulation cascade is initiated and fibrinolysis is inhibited by plasminogen activator inhibitor and other factors, favoring formation of a fibrin matrix (24). Fibroblasts that originate from the subpleural tissues (and perhaps in part from mesothelial-mesenchymal transition) migrate into the pleural space, infiltrate the expanding matrix, and secrete collagen and other extracellular matrix components (25). As the fibrotic process progresses, the parietal and visceral pleural surfaces fuse in a durable way that prevents future pneumothoraces or effusion.

We found that HAM particles instilled into the intrapleural space are at least as effective as talc at inducing a pleural reaction based on measures of pleural thickness, fibrosis, and adhesions, as well as elevation of serum and pleural lavage fluid markers of inflammation CRP (26), IL-6, TNF-α, and IL-1β. In addition, pleurodesis with HAM particles was as effective as talc and measurably more durable, with greater reduction in the volume of TTNP-induced pneumothoraces at all points tested through 3 months after instillation. Placement of both talc and HAM into the pleural space was associated with osteoclastic transformation of pleural monocytes, based on upregulated expression of signature osteoclast genes including *Acp5*, *Atp6v0d2*, and *Ctsk*, and elevation in pleural fluid TRAP5b. Interestingly, HAM produced a 3- to 5-fold greater response than talc in RANKL and OPG levels in bronchoalveolar lavage fluid, as might be expected based on the bone-like composition of HAM. Osteoclast differentiation was associated with almost complete clearance of HAM particles from the pleural space within 3 months based on marked reductions in HAM particle volumes by μCT, while talc particle volumes remained unchanged. Inhibition of osteoclastogenesis with an anti-RANKL antibody inhibited the clearance of HAM. Deleting CTSK reduced pleural adhesions due to HAM but not those due to talc, consistent with a role for this key matrix degrading enzyme of osteoclasts in pleural fusion.

Toxicities of talc include ARDS in 3%–9% of instances (7, 8, 11, 15–18), contamination with low levels of lead (4 μg/g) that preclude use in pregnant patients and children, persistent inflammation that has been associated with avid FDG uptake on PET scanning for decades (27), chronic pain (28), and excessive bleeding with subsequent thoracic surgical procedures (29). We conducted a number of experiments to explore the relative toxicities of talc vs. HAM in mice. Following intrapleural instillation, we found that both talc and HAM induced weight loss followed by a delay in age-dependent weight gain that was somewhat more pronounced for talc than HAM. The weight, activity levels, and food intake of mice that were challenged with intrapleural saline, HAM, and talc were similar at day 42. At 3 months after instillation, there was persistent FDG avidity in the thorax of mice treated with talc consistent with chronic inflammation that was not present in the HAM-treated animals. These data suggest that HAM has a more favorable toxicity profile in mice than talc, which is likely due in large part to the eventual clearance of HAM but not talc.

Dissemination of intrapleural talc is known to occur in both humans and animals, and safety of talc is widely reported to be particle size dependent (30–32). Cases of ARDS occur more commonly with talc preparations that contain small particles (<10 μm) (7, 8, 11, 15–18). We found a direct relationship between the size of HAM particles (5,10, and 24 μm) and the effectiveness of pleural adhesion formation, consistent with the greater surface area of smaller particles on a mg-by-mg basis. Smaller particles were also more likely to result in greater degrees of transient weight loss and dissemination, as has been observed with talc in multiple animal models. On a detailed histological survey, we found no evidence of significant inflammation or fibrosis in major organs or tissues outside of the pleural compartment.

This study has several limitations. Mouse models do not fully recapitulate the human pleural microanatomy and microenvironment. Pleural membranes in humans are thicker and more complex than those of mice, including superficial and deep elastic lamina and greater amounts of loose connective tissue (33, 34). Experiments to validate the safety and efficacy of hydroxyapatite pleurodesis in a larger animal species (swine) are underway. Pleurodesis experiments were only performed in male mice since they proved to be better able to tolerate the intrapleural instillation procedure, but the extent of pleural adhesion formation induced by HAM was similar at day 14 for both sexes (Supplemental Figure 12). The mice bearing malignant effusions lost excessive amounts of weight or died in less than 2 weeks, and long-term experiments were not possible with this model.

In summary, we find that particles composed of hydroxyapatite produce more durable pleural fusion than talc, without the safety concerns associated with a nondegradable mineral particle that contains lead (and in some cases asbestos), and which remains as a foreign body in the thorax for the lifetime of the individual. Hydroxyapatite particles induce transient inflammation, and they are degraded within weeks to calcium and phosphate and cleared by the kidneys without significant elevations in serum calcium and phosphate. Currently, despite being the most effective pleurodesis method, talc pleurodesis is typically reserved for malignant effusions because of the potential complications. If HAM pleurodesis proves to be as effective as talc but safer and better tolerated in the long term, it could well become a first line agent for additional pleurodesis indications. Finally, it is quite likely that all particles placed into the pleural space disseminate to some degree, albeit most often at a subclinical level, since the parietal pleura has lymphatic stomata with 10 μm orifices that provide unrestricted access to the lymphatic circulation. Our data suggest that HAM particles that disseminate are cleared over time, which is a more appealing property for a pleurodesis agent than a nondegradable particle that may persist and induce lasting local and remote tissue inflammation and damage.

## Methods

### Sex as a biological variable.

We studied both male and female mice. There were no sex differences in degree of pleural inflammation or adhesions induced by HAM (Supplemental Figure 12) but male mice were better able to tolerate the intrapleural instillation of particles, with lower procedural mortality compared with female mice (7.7% vs. 20%, respectively), likely due to their greater BW. All subsequent studies were done with male mice.

### Materials availability.

All stable and unique reagents generated in this study are available from the lead contact upon reasonable request.

### Experimental model details.

C57BL/6J mice were obtained from Jackson Laboratory (Bar Harbor, ME). *Ctsk*^–/–^ mice were obtained from EMMA (EM:10406 [B6;129-Ctsk<tm1Psa>/Ph]). For postmortem studies, mice were sacrificed by intraperitoneal injection of Euthasol (Henry Schein, Melville, NY). All animals were maintained in a specific pathogen–free facility and were handled according to a University of Cincinnati IACUC-approved protocol and NIH guidelines.

### Reagents.

A list of all reagents including sources and catalog numbers is included in Supplemental Table 3. Hydroxyapatite particles (nanoXIM•HAp202) were obtained from Fluidinova and from Matexcel (10 and 24 μm particles). Laboratory grade talc was from ThermoFisher and clinical grade talc (Steritalc) was a gift from Novatech. The endotoxin content of talc and HAM particles were measured and determined to be < 1.0 pg/μg of each reagent as determined using the LAL Chromogenic Endotoxin Quantitation Kit (Thermo Scientific, Rockford, IL) according to the manufacturer’s instructions.

### Intrapleural particle instillation.

Talc particles and HAM particles were autoclaved and dried at 100°C, suspended in 300 μL sterile saline, and loaded into the hub of a 23 gauge needle attached to a syringe without a plunger. The particles were introduced into the right pleural space by the method of Acencio et al. (35) in which the needle is advanced until the contents of the barrel are drawn into the pleural space by negative intrapleural pressure. After the procedure, the mice were wrapped in paper towels and placed in a supine position for 5 minutes. During this time, they were closely monitored until they had completely recovered. In models of malignant effusion, 5 × 10^4^ LL/2 cells (LLC1, ATCC) were instilled into the pleural space 4 days prior to intrapleural instillation of talc or HAM particles (35).

### Pleural thickness, fibrosis, and adhesion scoring.

Pleural thickness was quantified on Masson’s trichrome reagent–stained lung sections. Pleural adhesions were quantified according to the scale described by Xie et al. (36). Adhesion scores were determined for each plane of the thoracic cavity (ventral, lateral, dorsal, diaphragmatic, and mediastinal sides) for both the left and right lungs. The sum of these scores was used to calculate the total adhesion score.

### Postmortem TTNP and μCT.

Mice were sacrificed at the indicated time points after the intrapleural administration of HAM, talc, or saline. Pneumothorax was induced in postmortem mice by passes of a 20 gauge needle through both hemithoraces, 4 times each from the ventral, lateral, and diaphragmatic surfaces. μCT scans (Inveon, Siemens) were performed to estimate pneumothorax volume and particle volume on postmortem animals for terminal experiments. The Inveon Research Workplace (Siemens) general analysis and 3D Slicer were used for analysis. CT images were created from DICOM data by 3D Slicer.

### μPET/CT scanning.

Mice were placed in the supine position on an imaging platform under isoflurane anesthesia with continuous warming. 18F-FDG (11–18 MBq) was delivered by tail vein or retro-orbital injection, and imaging was performed 1 hour later on a μPET scanner over a 15-minute period (Siemens Inveon, Knoxville, TN). CT scan images (80 kVp, 500 μA, at 120 projections; approximately 6 minutes) were then acquired for anatomical reference overlay with PET images and for PET attenuation correction. CT images were reconstructed using the Feldkamp, Davis, and Kress (FDK) algorithm, and PET images were reconstructed by ordered subset expectation maximization in 2 dimensions (OSEM-2D). Histogramming and reconstruction with scatter correction were applied using Siemens software. Initial postprocessing was performed with Inveon Research Workplace for standardized uptake value (SUV) per gram BW. PET images were corrected for radioactive decay, injected dose and BW and expressed as SUV, with SUV = tissue activity (kBq/cc)/[injected dose (kBq)/BW (g)].

### Histology, IHC, and immunocytochemistry studies.

Mice were sacrificed and tissues were fixed with 10% buffered formalin phosphate, embedded in paraffin, and stained with H&E. For cytology and immunocytochemistry studies, pleural lavage cells were spun onto glass slides (700 rpm, 5 min) and dried overnight. The von Kossa technique was used to stain calcium deposits in the lung by application of 3% silver nitrate to the lung sections, exposure to strong light for 30 min, and counterstaining with Nuclear Fast Red 5 (Polysciences Inc.). The percentage of HAM particle positive sections of 100 sections surveyed was quantified; + = 1%–20%, ++ = 21%–50%, +++ = 51%–75%, ++++ = 76%–100%. Masson’s trichrome staining was used to identify collagen deposition (Newcomer Supply). For particles that were found within the parenchyma of tissues, we did not perform additional immunohistochemical studies to precisely localize them (e.g. LYVE staining to determine if particles were within lymphatics), and simply described them as within the ‘interior’. For TRAP staining, tissue sections were incubated with prewarmed (37°C) TRAP staining mix (University of Rochester Medical Center TRAP protocol) for 1 hr and counterstaining with Harris hematoxylin for 5 sec. IHC analysis was performed on formalin-fixed, paraffin embedded material. Sections (5 μm) were deparaffinized and rehydrated with dH2O. The sections were subjected to antigen retrieval with citrate buffer, blocked with normal serum, and probed with specific primary antibodies anti-cathepsin K (ab19027, polyclonal, 1:750) obtained from Abcam (Cambridge, UK), and anti-CALCR LS (LS-A769, polyclonal, 9 μg/mL) obtained from LS Bio (Seattle, WA) followed by horseradish peroxidase linked anti-rabbit secondary antibody (Cell Signaling Technology Inc., Beverly, MA). Slides were developed with DAB substrate, counter stained with hematoxylin, dehydrated, and mounted.

### Measurement of hydroxyproline content in the mouse lung.

For lung surface hydroxyproline measurements, the hearts and lungs of mice were excised as a block, and immersed in 5 mL of a 2 mg/mL solution of type I collagenase from Clostridium histolyticum (Thermo Scientific) in 50 mM Tris buffer at pH 7.42 containing 10 mM CaCl_2_ for 1 hr at 37°C (37). For whole lung hydroxyproline measurements, lung tissues were homogenized with 0.5 mL of dH2O per 100 mg of tissue. Equal volumes of 12 M HCl and collagenase supernatant or whole lung homogenate were mixed, and the samples were hydrolyzed at 95°C for 20 h and centrifuged at 13,000*g* for 10 min. The supernatant was collected and hydroxyproline in the supernatant was determined using Hydroxyproline Assay Kit (QuickZyme Biosciences, Netherlands), according to the manufacturer’s instructions.

### Pleural lavage and cell collection.

After euthanizing the mice, the sternum was carefully removed, ensuring not to damage the diaphragm. The lung cavity was flushed with 250 μL of saline and repeated for 20 times to collect approximately 5 mL per mouse. The pleural lavage fluid was centrifuged at 500*g* for 3 min to pellet the cells. Gene expression in pleural lavage cells was assessed by quantitative PCR (qPCR), and the cytokines in the supernatant from the first 1 mL of pleural lavage fluid were quantified by enzyme-linked immunosorbent assay (ELISA).

### Anti-RANKL monoclonal antibody treatment.

C57BL/6J mice were treated with i.p. anti-mouse RANKL mAb (Bio-X-Cell, catalog BE0191, clone IK 22-5) or a Rat IgG2a isotype Control IgG (Bio-X-Cell, catalog BE0089, clone 2A-3) as reported (12), before being sacrificed 28 days after challenge, followed by assessments of particle density by μCT.

### Preparation of RNA and qPCR.

Total RNA was isolated from pleural lavage fluid cell pellet and murine whole lung tissues using RNAzol RT (Molecular Research Center, Cincinnati, OH), according to the manufacturer’s instructions. cDNA was synthesized using the High-Capacity cDNA Reverse Transcription Kit (Applied Biosystems, Waltham, MA). Primers used are listed in Supplemental Table 4. RT-qPCR was performed using a SYBR Green Master Mix (Applied Biosystems) with primer pairs for fibrosis-related and osteoclast-related genes, as well as β-actin (Actb) as internal controls.

### ELISA.

The levels of analytes in mouse pleural lavage fluid and serum were measured by ELISA as follows: TRAP5b, Mouse TRAPTM (TRAP5b) ELISA Kit (Immunodiagnostic Systems, United Kingdom); IL-1β, Mouse IL-1β DuoSet ELISA Kit (R&D Systems Inc); CRP, Mouse C-Reactive Protein DuoSet ELISA Kit (R&D Systems Inc); RANKL, Mouse TRANCE/RANKL/TNFSF11 Quantikine ELISA Kit (R&D Systems Inc); OPG, Mouse Osteoprotegerin/TNFRSF11B DuoSet ELISA Kit (R&D Systems Inc); M-CSF, Mouse M-CSF DuoSet ELISA Kit (R&D Systems Inc) according to the manufacturer’s instructions.

### Food intake.

Mice were individually housed and acclimated to the indirect calorimetry cages for one day before data collection began. Indirect calorimetry was conducted using the PhysioScan open-circuit Oxymax system (version 5.35), part of the Comprehensive Laboratory Animal Monitoring System (CLAMS; Columbus Instruments, Columbus, OH, USA). Rodent diet was placed in each food hopper, which is equipped with sensitive scales to detect even slight changes in food weight. The system automatically measured and recorded daily food intake as the mice consumed food from the hoppers

### Locomotive activity.

The activity of individually housed mice was evaluated on a relative, not absolute, basis using an 8-cage rack OPTO-M3 Sensor system (Columbus Instruments, Columbus, OH, USA). Home cages were placed in Smart Frame stainless steel cage rack frames (Hamilton-Kinder, Poway, CA, USA). Infrared photobeam interruption sensors mounted in the frames detected each animal’s movements. Activity counts (beam interruptions) were recorded every 60 min throughout the light and dark cycles.

### Statistics.

Statistical analyses were performed using GraphPad Prism 10 (version 10.4.0, GraphPad Software). Differences between 2 groups were compared using the 2-tailed unpaired *t* test with the Welch correction. In experiments in which more than 2 groups were involved, 1-way ANOVA was used followed by Tukey’s method for multiple-comparison test. Differences were considered significant at *P* < 0.05.

### Data availability.

Values for all data points in graphs are reported in the Supporting Data Values file.

### Study approval.

The animal experimental procedures were approved by the IACUC at the University of Cincinnati and conformed to guidance provided in the *Guide for the Care and Use of Laboratory Animals* (National Academies Press, 2011).

## Author contributions

FXM developed the concept, designed experiments, analyzed data, and wrote the manuscript. Y Tanaka, Y Takahashi, and YS designed and performed experiments, analyzed data, and wrote the manuscript. LBP performed histological and immunohistochemical experiments. SLT and WZ analyzed data and provided osteoclast expertise. JCW assisted with the design and interpretation of quantitative μCT experiments. XW helped with μCT and CT PET imaging. KAWB provided interpretation for pathological and toxicologic specimens. All coauthors read and edited the manuscript. The order of co–first authors (Tanaka, Takahashi) was determined by the chronology and relative extent of contributions, with Tanaka initiating the project and leading the experimental work.

## Supplementary Material

Supplemental data

Supporting data values

## Version 1 08/26/2025

In-Press Preview

## Version 2 10/08/2025

Electronic publication
