# Supplementary material for: Hydroxyapatite microspheres induce durable pleurodesis and are rapidly cleared by pleural osteoclasts
Source: JCI Insight. 2025 Aug 21;10(19):e192981. doi: 10.1172/jci.insight.192981 (PMC12513485; doi:10.1172/jci.insight.192981)
Supplement: Supplemental data [file jciinsight-10-192981-s102.pdf]

Supplemental contents

Supplemental Fig. 1-Scanning electron microscopy of particles used in the study

Supplemental Fig. 2-Pleural fibrosis after intrapleural particle challenge

Supplemental Fig. 3-Inflammatory responses to intrapleural particle challenge

Supplemental Fig. 4-Effectiveness of pleurodesis for HAM and talc

Supplemental Fig. 5-Body weight after pleurodesis for malignant pleural effusion

Supplemental Fig. 6-Clearance of particles after intrapleural instillation of HAM and talc.

Supplemental Fig. 7-Serum levels of calcium and phosphate after intrapleural particle challenge

Supplemental Fig. 8-Physiologic effects of pleurodesis

Supplemental Fig. 9-Size dependence of intrapleural HAM associated weight loss and dissemination.

Supplemental Fig. 10-Dose dependence of intrapleural HAM associated wt loss, adhesion formation and dissemination formation.

Supplemental Fig. 11-Time dependent clearance of disseminated HAM particles.

Supplemental Fig. 12-Sex differences in body weight change and pleurodesis scores.

Supplemental Table1-HAM and talc particle sizes measured by scanning electron microscopy

Supplemental Table 2-HAM and talc dose calculations

Supplemental Table 3–Reagents used

Supplemental Table 4-Primers used for qRT-PCR

Supplemental Fig. 1.

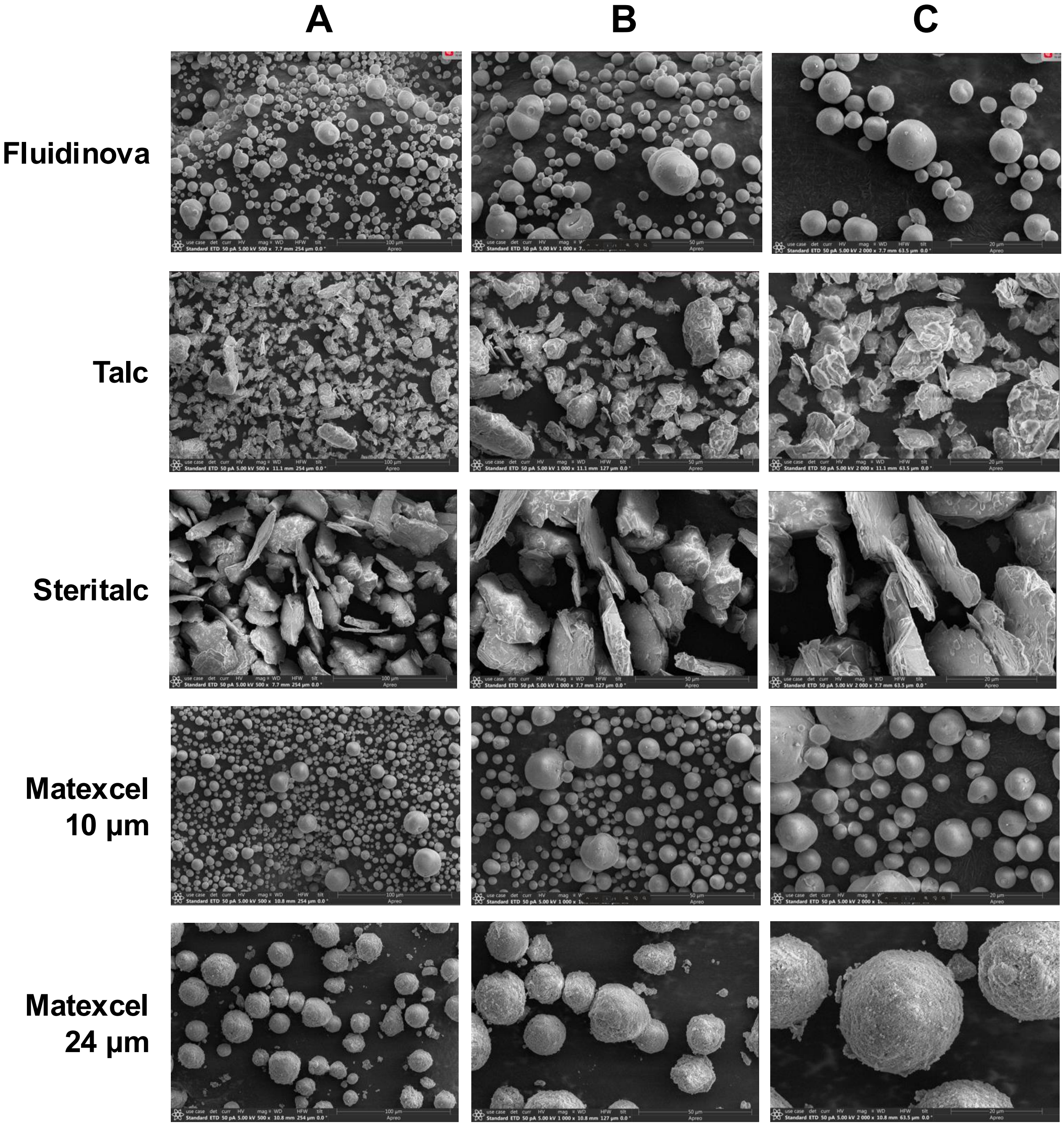

Supplemental Fig. 1. Scanning electron microscopy of particles used in the study. Scale bars of columns A,B, and C are 100 µm, 50 µm and 20 µm, respectively.

Supplemental Figure 2(corresponds to Fig 1)

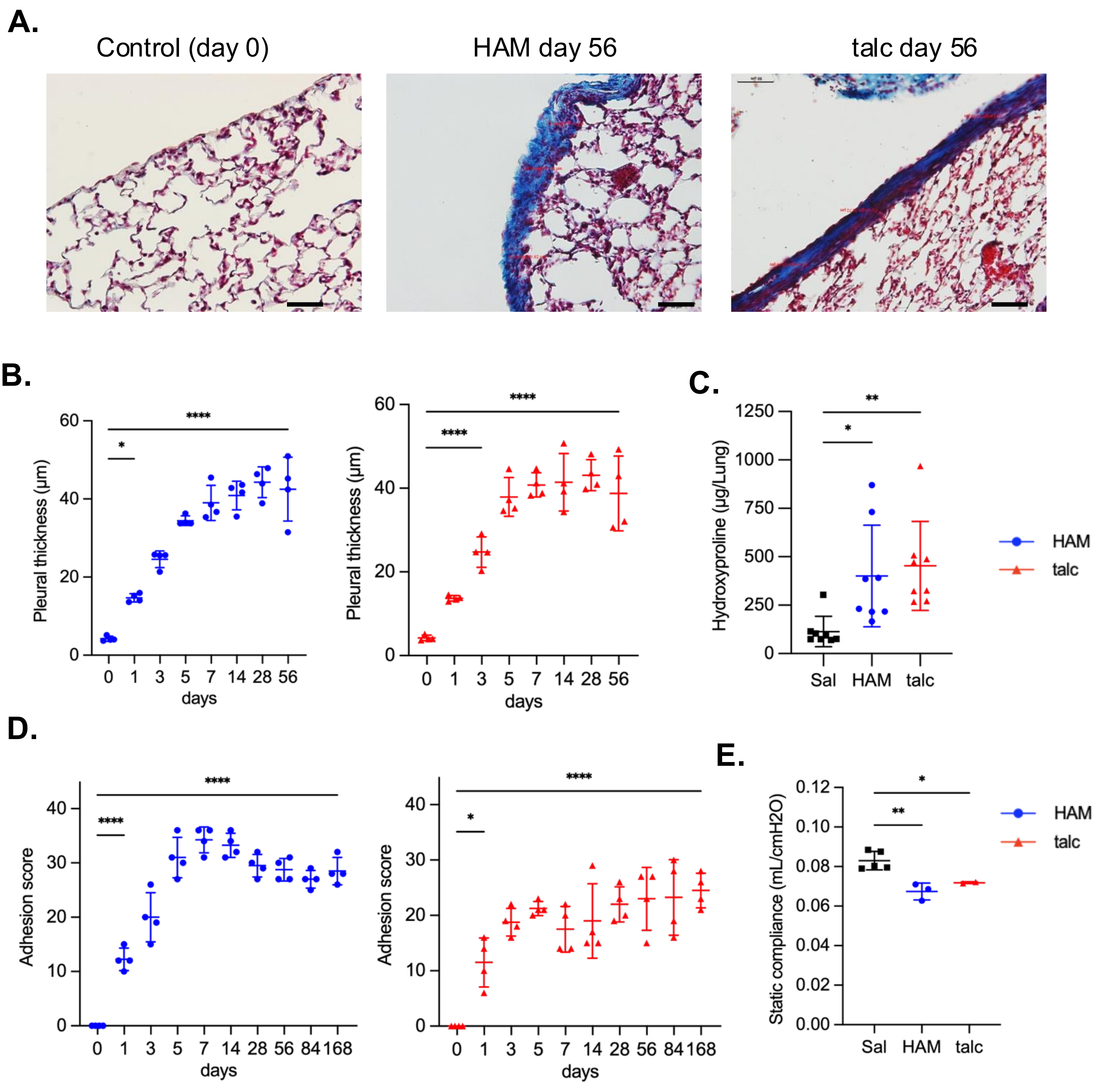

Supplemental Fig. 2-Pleural fibrosis after intrapleural particle challenge. A. Masson's Trichrome stained sections of lungs harvested from control mice and at day 56 after intrapleural challenge with HAM or talc. Shown at low magnification to demonstrate pleural fibrosis and the absence of parenchymal fibrosis. (Scale bar, 50 µm) **B.** Pleural thickness after HAM (blue) or talc (red) particle challenge in individual mice corresponding to Fig. 1D. **C.** Whole lung hydroxyproline from particle challenged mice, corresponding lung surface hydroxyproline in Fig. 1E. **D.** Adhesion scores after HAM (blue) or talc (red) pleurodesis, corresponding to Fig. 1F. **E.** Static compliance at day 56 post HAM or talc pleurodesis. Comparisons were by by one-way analysis of variance (ANOVA) followed by Tukey's method for multiple group comparisons. Data are mean ± SD. *P < 0.05, **P < 0.01, ***P < 0.001 and ****P < 0.0001.

Supplemental Figure 3 (corresponds to Fig 2)

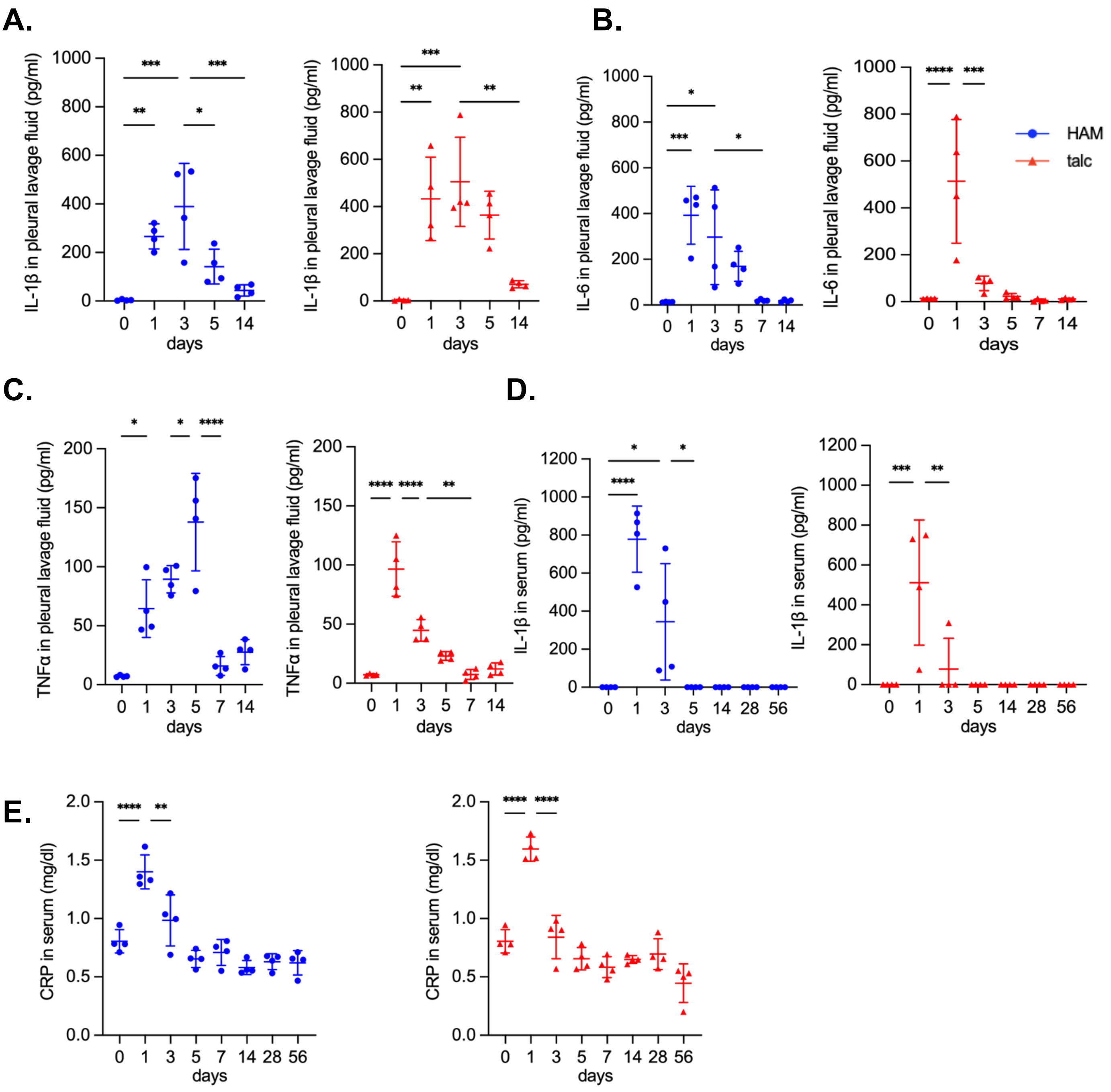

Supplemental Fig. 3. Inflammatory responses to intrapleural particle challenge. (A-C) Pleural lavage fluid and **(D,E)** serum cytokines from individual mice treated with intrapleural HAM and talc, corresponding to Figs. 2A-C and 2 D-E, respectively. Data are mean ± SD. Comparisons were by one-way analysis of variance (ANOVA) followed by Tukey's method for multiple group comparisons. **P* <0.05, ***P* < 0.01, ****P* < 0.001 and *****P* < 0.0001.

Supplemental Figure 4 (corresponds to Fig 5)

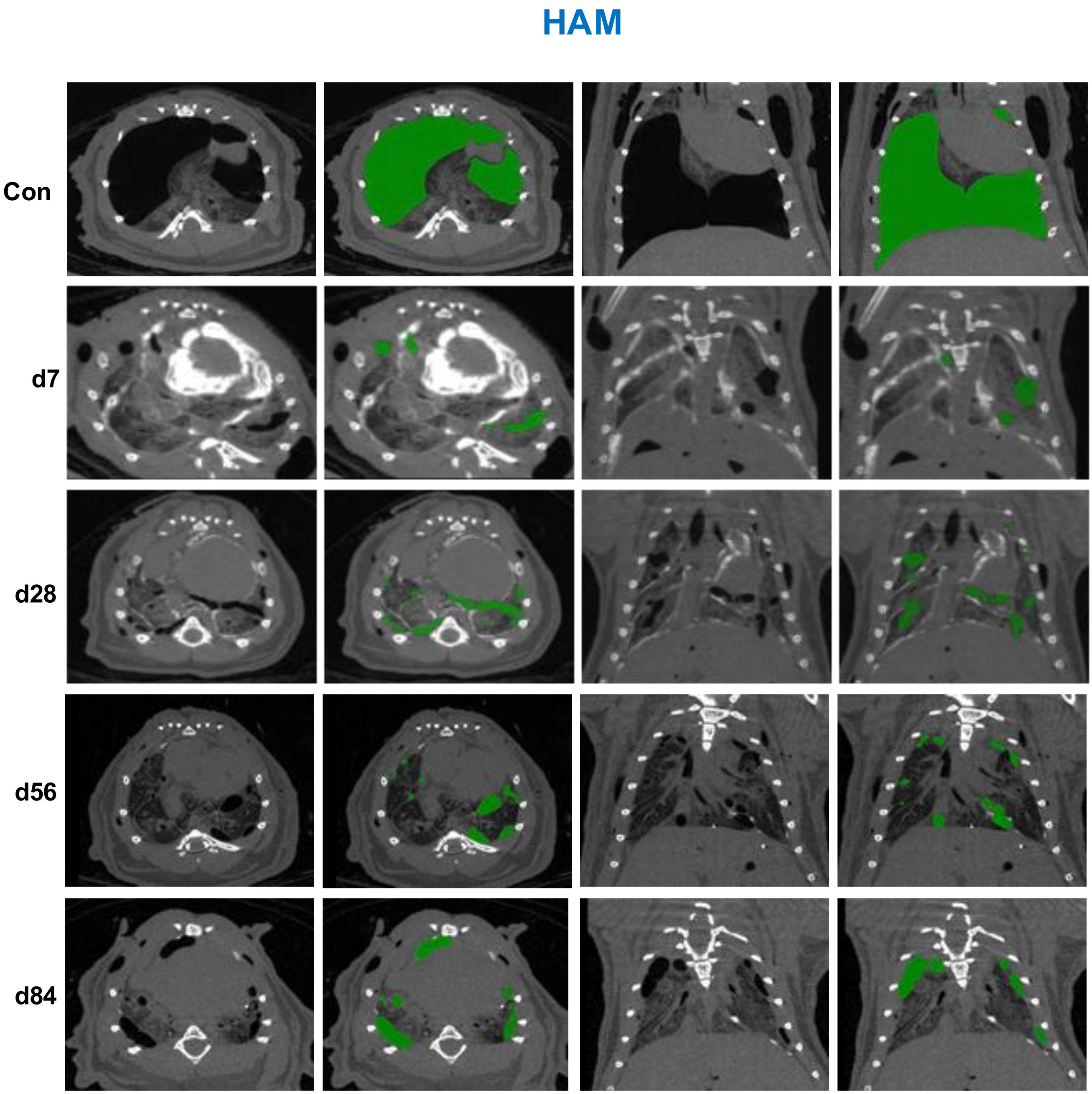

Supplemental Fig. 4A. Effectiveness of HAM pleurodesis. Axial and coronal unadulterated and companion colorized (to highlight intrapleural air) microCT images obtained at the indicated time points after intrapleural HAM administration, and following postmortem TTNP to induce pneumothorax. Corresponds to Figure 5.

Supplemental Figure 4 (corresponds to Fig 5)

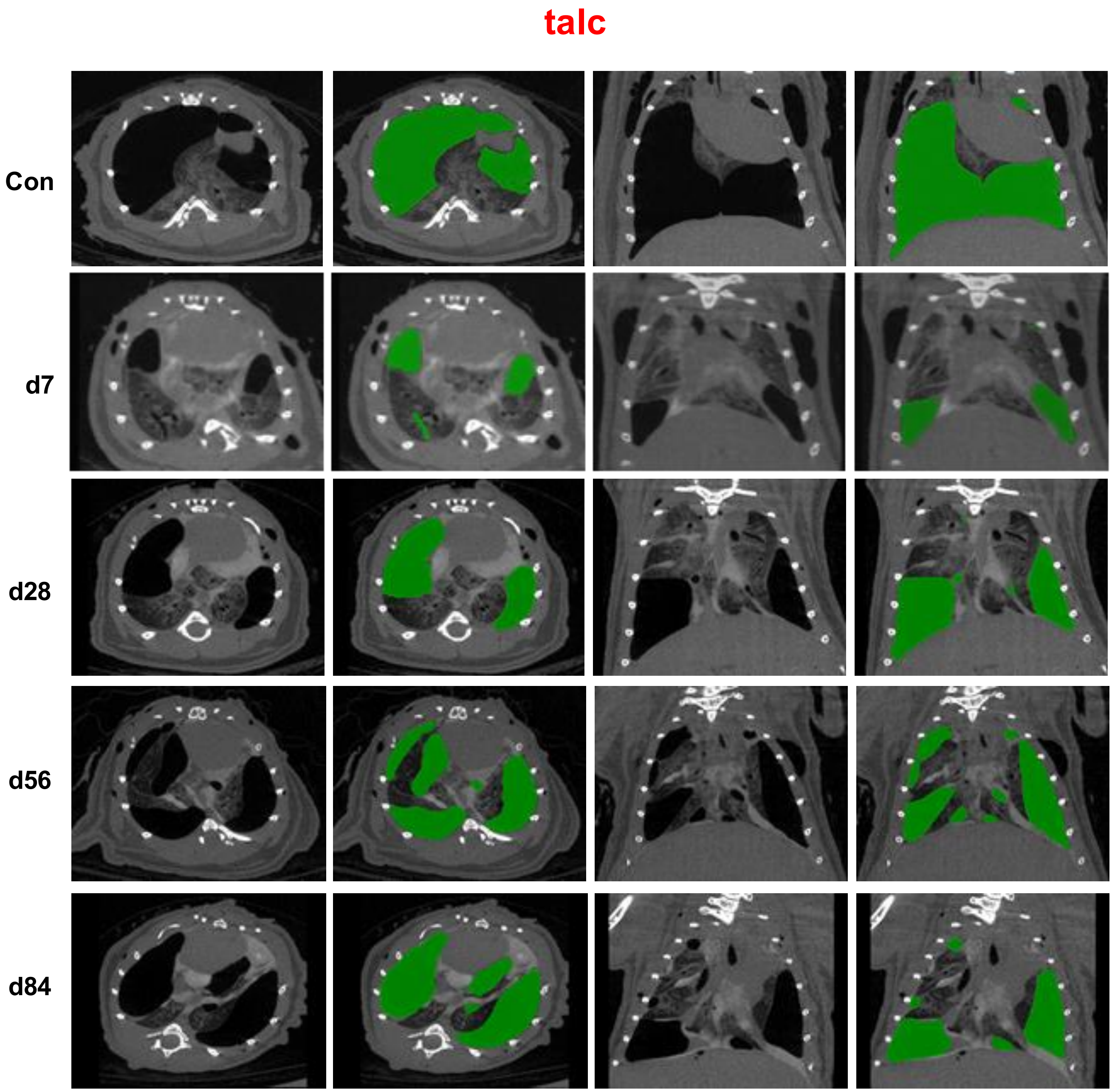

Supplemental Fig 4B. Effectiveness of talc pleurodesis. Axial and coronal unadulterated and companion colorized (to highlight intrapleural air) microCT images obtained at the indicated time points after intrapleural talc administration, and following postmortem TTNP to induce pneumothorax. Corresponds to Figure 5.

Supplemental Figure 5 (corresponds to Fig 6)

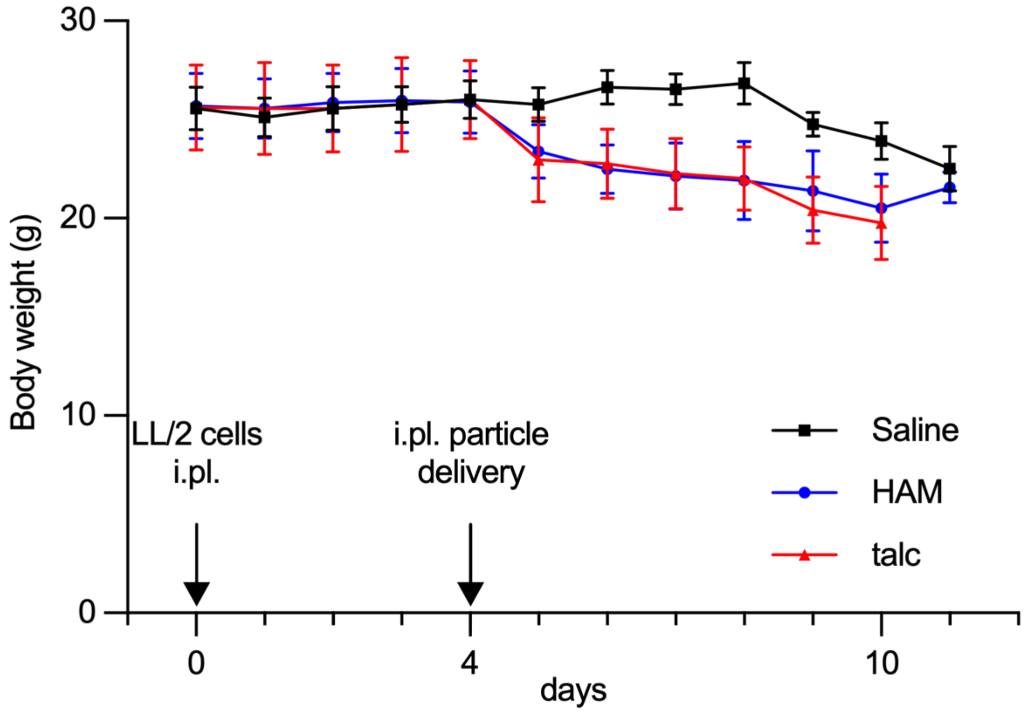

Supplemental Fig 5. Body weight after pleurodesis for malignant pleural effusion. Change in body weight (in grams) over time after intrapleural instillation of LL/2 cells followed by HAM or talc, corresponding to Figure 6. Data are mean ± SD.

Supplemental Figure 6A (corresponds to Fig 7)

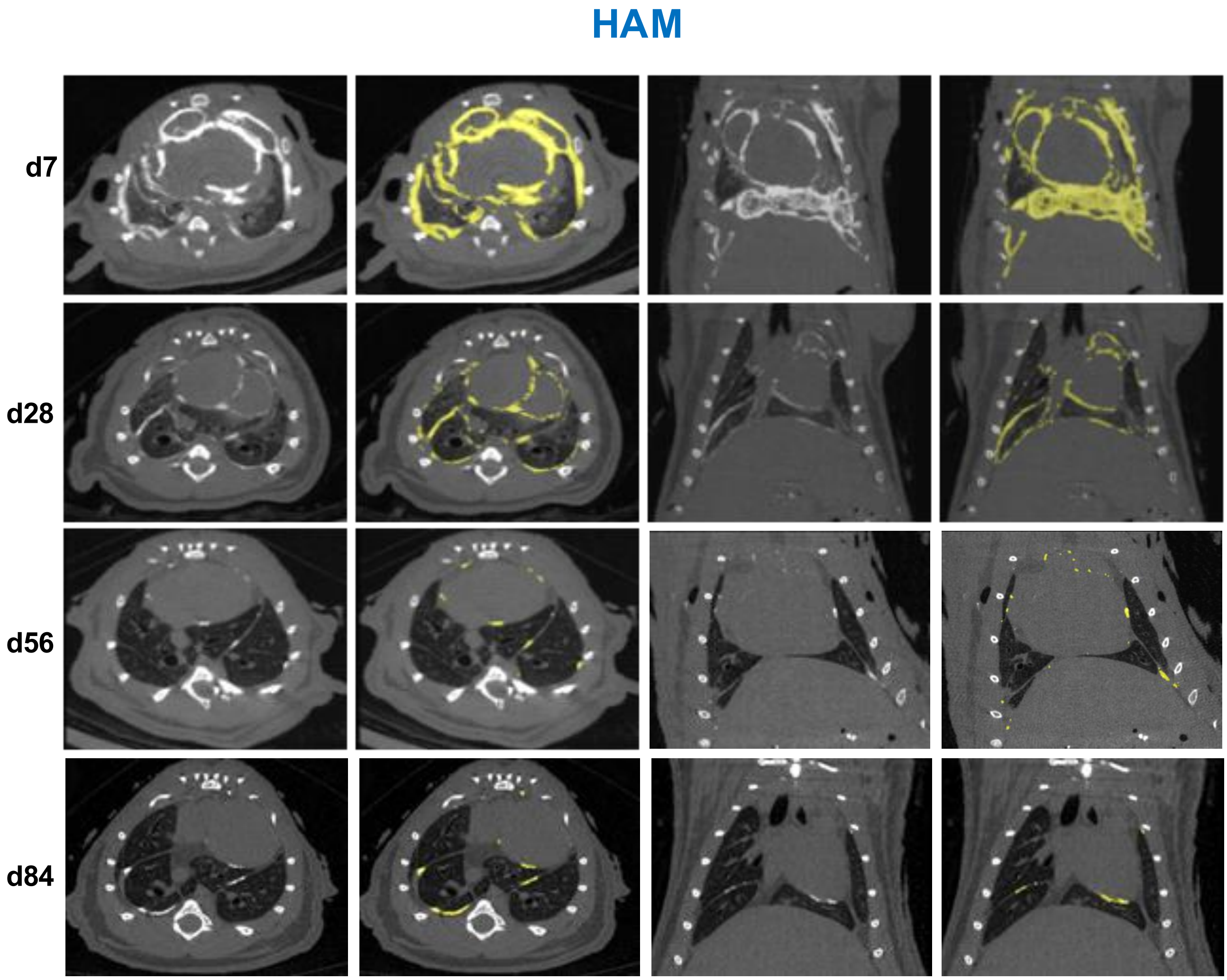

Supplemental Fig 6A. Clearance of HAM particles after intrapleural instillation. Axial and coronal unadulterated and companion colorized (to highlight HAM particles) microCT images obtained at the indicated time points after intrapleural HAM administration, corresponding to Figure 7.

Supplemental Figure 6B (corresponds to Fig 7)

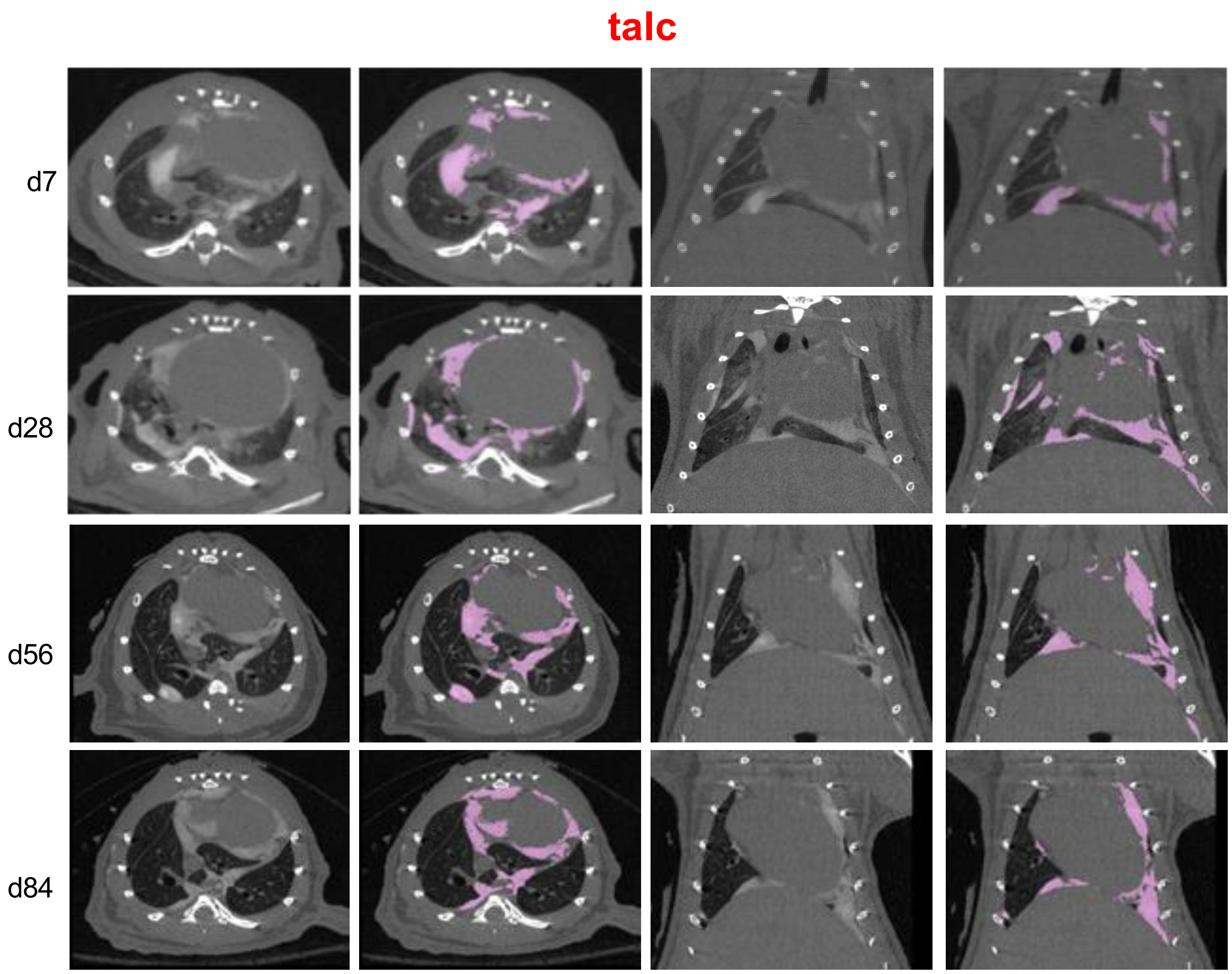

Supplemental Fig. 6B. Clearance of talc particles after intrapleural instillation. Axial and coronal unadulterated and companion colorized (to highlight talc particles) microCT images obtained at the indicated time points after intrapleural talc administration, corresponding to Figure 7.

Supplemental Figure 7

A.

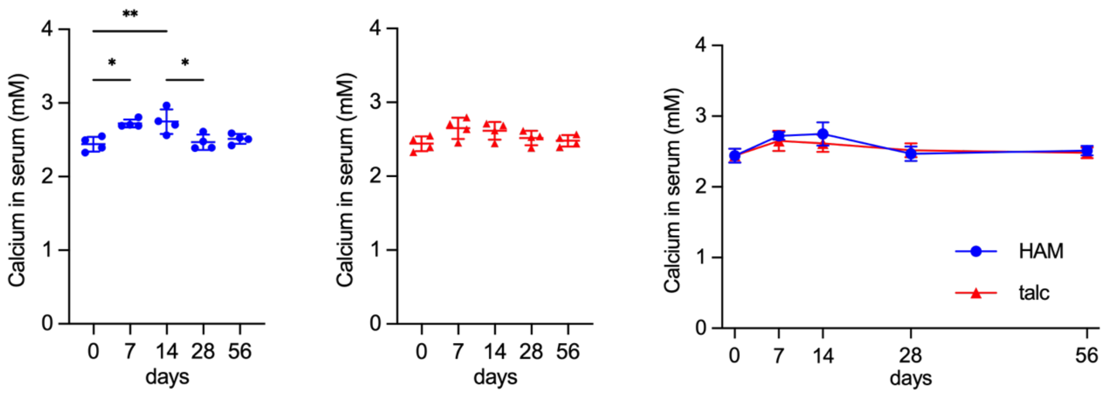

B.

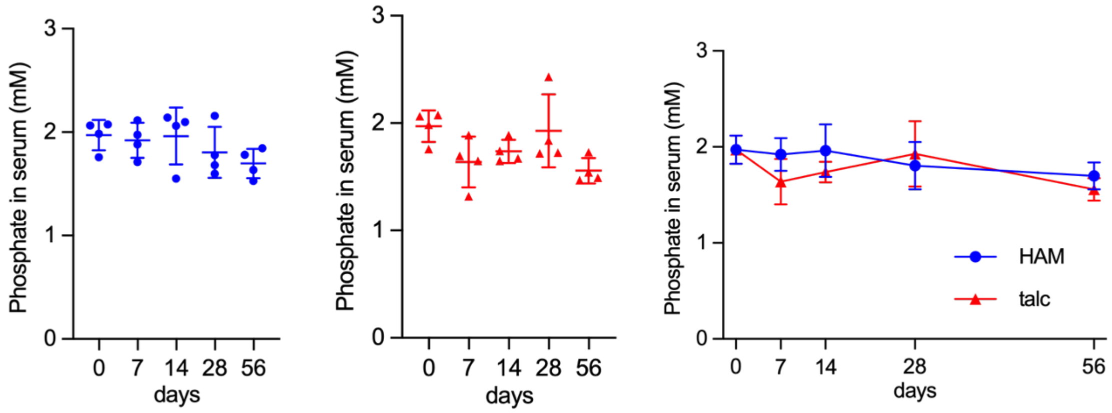

Supplemental Fig. 7. Serum levels of calcium and phosphate after intrapleural particle challenge. Serum calcium **(A)** and phosphate **(B)** levels were measured at the indicated time points after intrapleural treatment with HAM and talc. Data are mean ± SD. Comparisons were by one-way analysis of variance (ANOVA) followed by Tukey's method for multiple group comparisons. *P < 0.05 and **P < 0.01

Supplemental Figure 8

A.

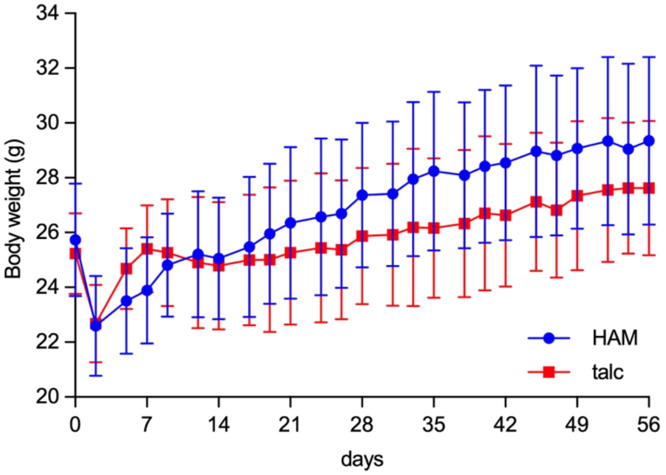

B.

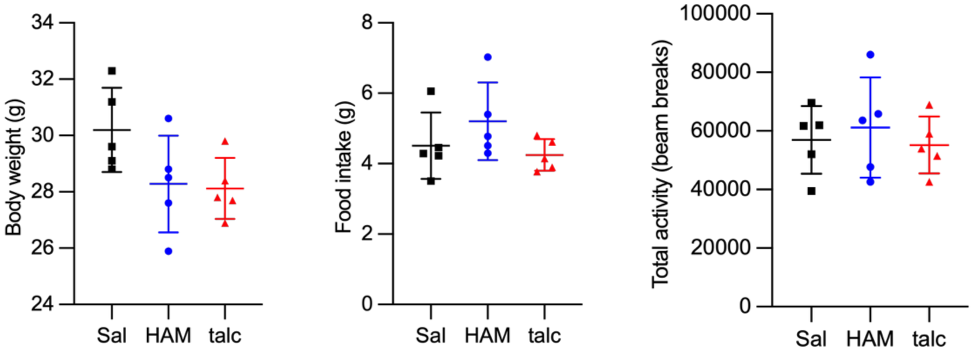

Supplemental Fig. 8. Physiologic effects of pleurodesis. (A) Mice weights were obtained 3 times per week over a 56d period after intrapleural challenge with HAM or talc. **(B)** On day 42, weights, food intake and total activity were compared by one-way analysis of variance (ANOVA) followed by Tukey's method for multiple group comparisons. *P < 0.05. Data are mean ± SD. There were no significant between differences in body weight, food intake or activity levels at day 42.

Supplemental Figure 9.

A.

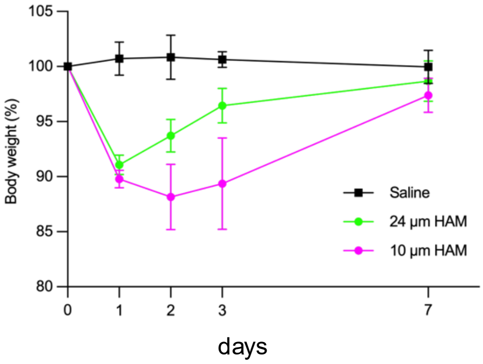

B.

	10 µm, 80 mg/mouse, Day 7 (Mouse No.1/No. 2/No. 3/No.4)	24 µm, 80 mg/mouse, Day 7 (Mouse No.1/No. 2/No. 3)
Lung	Surface: ++++/++++/++++/++++<br>Interior: -/++/-/+	Surface: ++++/++++/++++<br>Interior: +/+/+
Heart	Surface: ++++/+++/++++/++++<br>Intravascular: ++/+/+/+<br>Interior: -/-/+/+	Surface: +/++++/++++<br>Intravascular: -/-/+<br>Interior: -/++/++
Chest wall	Surface: ++++/++++/++++/++++<br>Interior: ++/++/++/+	Surface: +++/++++/++++<br>Interior: -/-/+
Diaphragm	Surface: ++++/++++/++++/++++<br>Interior: -/-/-/-	Surface: ++++/++++/++++<br>Interior: -/-/-
Liver	Surface: -/-/-/+<br>Interior: -/-/-/-	Surface: ++/-/-<br>Interior: -/-/-
Spleen	Surface: -/-/+/-<br>Interior: -/-/-/-	Surface: ++++/+/+/-<br>Interior: -/-/-
Kidney	Surface: -/-/+/-<br>Interior: -/+/-/+	Surface: +/-/-<br>Interior: -/-/-
Peritoneum	Surface: +/-/-/+<br>Interior: -/-/-/+	Surface: ++++/-/-<br>Interior: -/-/-
Brain	Surface: -/-/-/-<br>Interior: -/-/-/-	Surface: -/-/-<br>Interior: -/-/-

C.

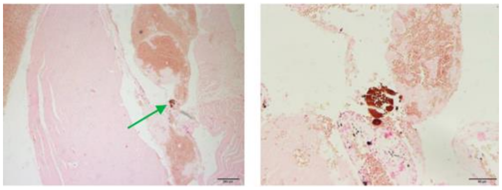

D.

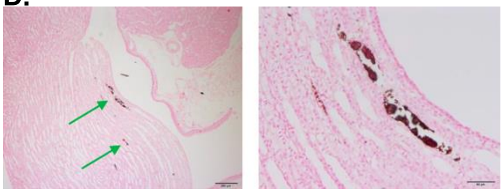

Supplemental Fig. 9. Size dependence of intrapleural HAM associated weight loss and dissemination. Intrapleural 10 and 24 µm HAM particles or saline were delivered at time zero and **(A)** weights were obtained at baseline, and days 1,2,3 and 7. Data are mean ± SD. **(B)** On day 7, animals were sacrificed and tissues were harvested for von Kossa staining. The percentage of HAM particle positive sections of all sections surveyed for each mouse was quantified. + = 1-20%, ++ = 21-50%, +++ = 51-75%, ++++ = 76-100%. (**C-D)** Day 7 low power (left panels) and high power (right panels) images of intravascular particles in the heart **(C)** and intraparenchymal particles in the kidney **(D)** demonstrated by von Kossa staining. Corresponds to summary Figure 9.

Supplemental Figure 10

A.

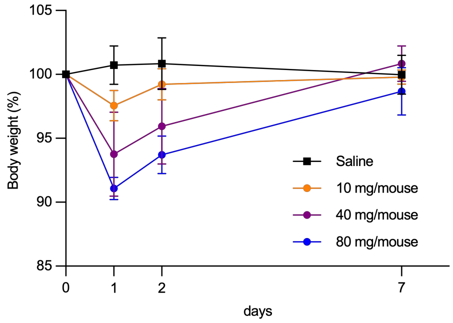

B.

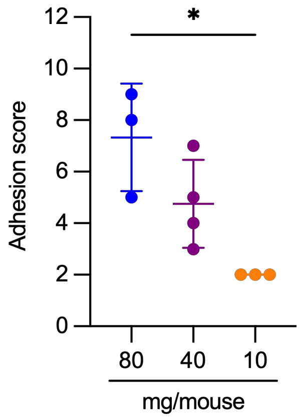

C.

	24 µm, 40 mg/mouse, Day 7 (Mouse No.1/No. 2/No. 3/No.4)	24 µm, 80 mg/mouse, Day 7 (Mouse No.1/No. 2/No. 3)
Lung	Surface: ++++/++++/++++/++++<br>Interior: ++/-/-/-	Surface: ++++/++++/++++<br>Interior: +/+/+
Heart	Surface: -/+++/++++/++<br>Intravascular: -/-/-/-<br>Interior: -/-/++/-	Surface: +/++++/++++<br>Intravascular: -/-/+<br>Interior: -/++/++
Chest wall	Surface: ++++/+++/+/+++<br>Interior: -/++/-/+	Surface: +++/++++/++++<br>Interior: -/-/+
Diaphragm	Surface: ++++/++++/++++/++++<br>Interior: -/-/-/-	Surface: ++++/++++/++++<br>Interior: -/-/-
Liver	Surface: -/-/-/<br>Interior: -/-/-/	Surface: ++/-/-<br>Interior: -/-/-
Spleen	Surface: -/-/-/-<br>Interior: -/-/-/-	Surface: ++++/+/-<br>Interior: -/-/-
Kidney	Surface: -/-/-/-<br>Interior: -/-/-/-	Surface: +/-/-<br>Interior: -/-/-
Peritoneum	Surface: -/-/-/-<br>Interior: -/-/-/-	Surface: ++++/-/-<br>Interior: -/-/-
Brain	Surface: -/-/-/-<br>Interior: -/-/-/-	Surface: -/-/-<br>Interior: -/-/-

Supplemental Fig. 10. Dose dependence of intrapleural HAM associated weight loss, adhesion formation and dissemination. Intrapleural 24 µm HAM particles at low (10 mg/mouse), medium (40 mg/mouse) and high doses (80 mg/mouse) or saline (black circle) were delivered to mice at time zero and (A) weights were obtained at baseline, and days 1,2,3 and 7. (B) On day 7, animals were sacrificed and adhesion scores were determined as outlined in Fig 1, and (C) tissues were harvested for von Kossa staining and scoring for the presence of particles. The percentage of HAM particle positive sections of all survey sections surveyed for each mouse was quantified. + = 1-20%, ++ = 21-50%, +++ = 51-75%, + +++ = 76-100%. Data are mean ± S.D. *$P < 0.05$.* Corresponds to summary Figure 9.

Supplemental Figure 11

A.

	24 μm, 80 mg/mouse, Day 7 (Mouse No.1/No. 2/No. 3)	24 μm, 80 mg/mouse, Day 28 (Mouse No.1/No. 2/No. 3/No.4)	24 μm, 80 mg/mouse, Day 84 (Mouse No.1/No. 2/No. 3/No.4)
Lung	Surface: ++++/++++/++++<br>Interior: +/+/+	Surface: ++++/++++/++++/++++<br>Interior: -/+/-/+	Surface: ++++/++++/++++/++++<br>Interior: ++/-/++/-
Heart	Surface: +/++++/++++<br>Intravascular: -/-/+<br>Interior: -/++/++	Surface: +++/++++/++++/++++<br>Intravascular: -/-/-/-<br>Interior: -/++/-/-	Surface: +++/++++/++/++<br>Intravascular: -/-/-/-<br>Interior: -/+++/-/-
Chest wall	Surface: +++/++++/++++<br>Interior: -/-/+	Surface: ++++/++++/++++/++++<br>Interior: -/++/-/-	Surface: ++/-/++/++<br>Interior: ++/-/++/-
Diaphragm	Surface: ++++/++++/++++<br>Interior: -/-/-	Surface: ++++/++++/++++/++++<br>Interior: -/-/++/-	Surface: ++++/++++/++++/++++<br>Interior: ++/+/-/-
Liver	Surface: ++/-/-<br>Interior: -/-/-	Surface: -/-/-/-<br>Interior: -/-/-/-	Surface: -/-/-/-<br>Interior: -/-/-/-
Spleen	Surface: ++++/+/-<br>Interior: -/-/-	Surface: -/-/-/-<br>Interior: -/-/-/-	Surface: -/-/-/-<br>Interior: -/-/-/-
Kidney	Surface: +/-/-<br>Interior: -/-/-	Surface: +/-/-/-<br>Interior: -/-/-/-	Surface: -/-/-/-<br>Interior: -/-/-/-
Peritoneum	Surface: ++++/-/-<br>Interior: -/-/-	Surface: /-/-/-<br>Interior: /-/-/-	Surface: /-/-/-<br>Interior: /-/-/-
Brain	Surface: -/-/-<br>Interior: -/-/-	Surface: -/-/-/-<br>Interior: -/-/-/-	Surface: -/-/-/-<br>Interior: -/-/-/-

B.

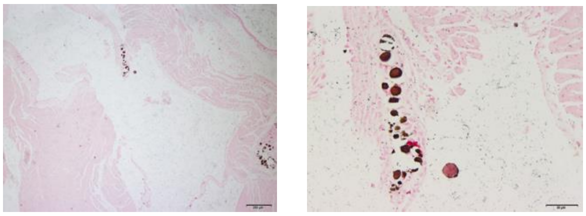

D.

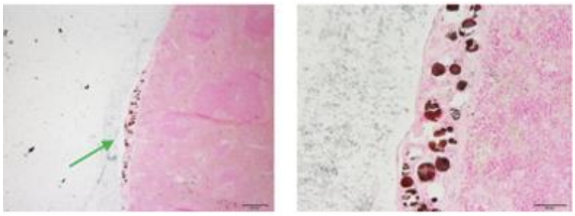

C.

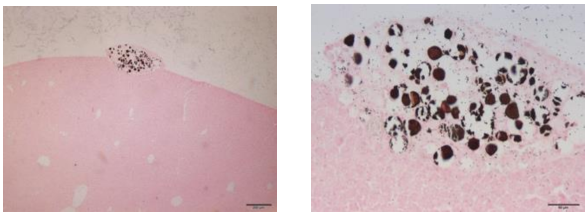

E.

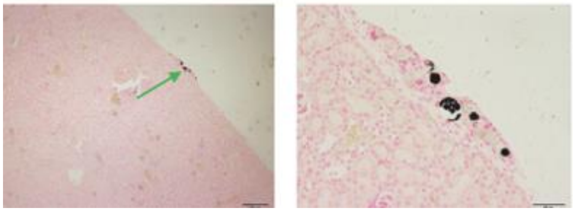

Supplemental Fig. 11. Time dependent clearance of disseminated HAM particles. (A) Intrapleural 24 μm HAM particles were delivered at 80 mg/mouse at time zero. **(A)** On day 7, 28 and 84, animals were sacrificed and tissues were harvested for von Kossa staining. The percentage of HAM particle positive sections of all sections was quantified. + = 1-20%, ++ = 21-50%, +++ = 51-75%, ++++ = 76-100%. (B-E) Day 7 images of intravascular particles in the heart (B) and particles on the surface of the liver (C), spleen (D) and kidney(E) are shown.

Supplemental Figure 12

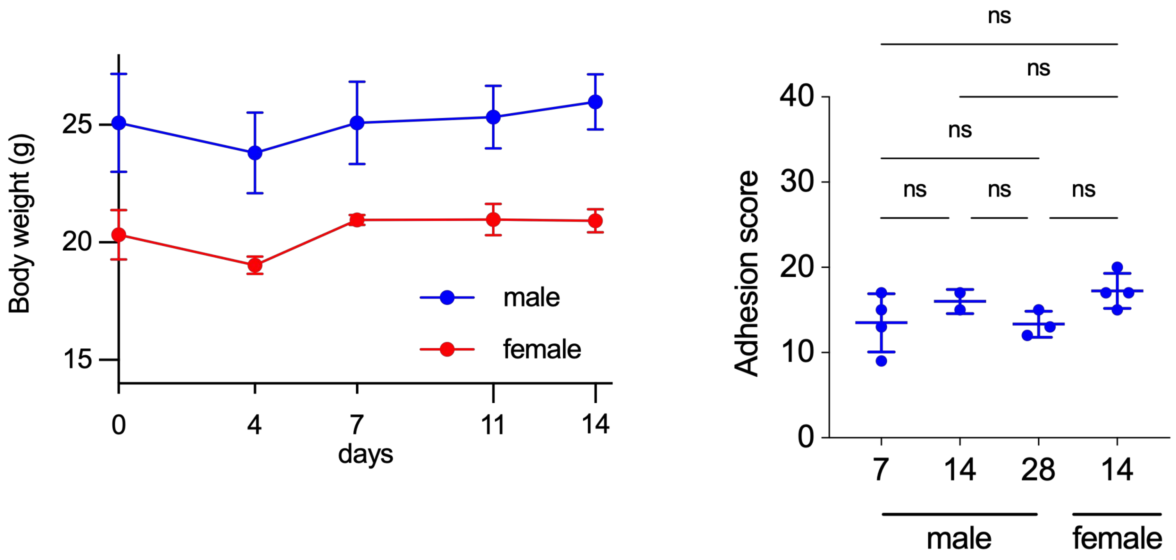

Supplemental Fig. 12. Sex differences. Body weight and pleural adhesion formation over time after intrapleural instillation (Fluidinova) HAM particles in male (n= 6) and female (n = 4) mice. Data are mean ± SD., p = ns

Supplemental Table 1.

Reagent	min	max	mean	S.D.
Laboratory talc	4.2	15.3	8.2	2.2
Fluidinova HAM	1.9	17.1	6.0	2.7
Steritalc	22.9	60.5	37.1	8.4
Matexcel HAM 10 µm	7.4	28.8	13.6	3.5
Matexcel HAM 24 µm	12.6	35.9	24.8	4.4

NIH Image J was used to estimate measures particle diameters on scanning electron microscopy images of 100 random particles of each type. Data are expressed in microns (µm).

Supplemental Table 2.

Species	Body wt (mg)	BSA (m^2)	Particle dose (mg)	Particle dose (mg/gm)	Particle dose (mg/m^2)
human	70,000	1.730	10000	0.142	5780
mouse	20	.007	40	2.0	5714

Particle dose calculation-The dose of talc used in mice (40 mg) was based on the maximum recommended human dose (10 grams) on the Steritalc label corrected for body surface area (1.73 m^2 for humans vs .007 m^2 for mice).

The dosage selection of 2 mg/g body weight (BW) for talc and 4 mg/g BW for nanoXIM-HAp202 (HAM) was based on (i) previously reported doses in the murine pleurodesis model, (ii) differences in particle shape, effective surface area, and formula weight.

Talc ($Mg_3Si_4O_{10}(OH)_2$) forms plate-like, layered crystals; milling exfoliates these layers and markedly increases the external surface. Medical-grade talc typically exhibits an external specific surface area (SSA) of roughly 7–25 m^2/g. In mice, up to 4 mg/g BW has been used in pleurodesis studies as the experimental dose, but both the literature and our pilot study showed that 2 mg/g BW reproducibly induces pleurodesis while maintaining an acceptable safety margin; this dose was therefore adopted.

The HAM particles we use consists of nearly spherical, dense microspheres. The supplier lists a BET SSA of ≥ 80 m^2/g for nanoXIM-HAp202, but this value includes internal mesopores accessible to nitrogen gas. Only the particle exterior interacts with pleural cells; for 5–10 µm spheres the geometric external SSA is only ~0.2–0.4 m^2/g—over an order of magnitude lower than the external SSA of layered talc. In addition, HAM has a molecular weight about 2.9-fold higher than talc, so the number of particles per unit mass is about~40 % of that of talc. To compensate for both the lower external surface and the lower particle number, the HAM mass was doubled, to 4 mg/g BW.

With these adjustments, HAM at 4 mg/g elicited acute inflammatory responses comparable to talc at 2 mg/g and produced more durable adhesions at 12 weeks.

Collectively, the mass ratio of talc 2 mg/g to HAM 4 mg/g is justified by physicochemical principles and corroborated by the experimental outcomes.

Supplemental Table 3.

Reagent	Form	Catalog #	Company	Location
TRAP 5b	Mouse TRAPTM (TRAP 5b) ELISA Kit	Cat #: SB-TR103	Immunodiagnostic Systems	United Kingdom
IL-1β	Mouse IL-1 beta DuoSet ELISA Kit	Cat #: DY40105	R&D Systems Inc	Minneapolis, MN
CRP	Mouse C-Reactive Protein DuoSet ELISA Kit	Cat #: DY1829	R&D Systems Inc	Minneapolis, MN
RANKL	Mouse RANK L/TNFSF11 Quantikine ELISA Kit	Cat #: MTR00	R&D Systems Inc	Minneapolis, MN
OPG	Mouse OPG/TNFRSF11B ELISA Kit	Cat #: DY459	R&D Systems Inc	Minneapolis, MN
M-CSF	Mouse M-CSF DuoSet ELISA Kit	Cat #: DY416	R&D Systems Inc	Minneapolis, MN
anti-mouse RANKL	Monoclonal antibody	Cat #:BE0191, Clone IK 22-5	Bio-X-Cell	West Lebanon, NH
Rat Ig2a	Isotype control IgG	Cat #: BE0089, Clone 2A-3	Bio-X-Cell	West Lebanon, NH
anti-Rabbit IgG	Secondary antibody	Cat # 7074V	Cell Signaling	Danvers, MA
Hydroxyproline	Assay kit	Cat #: QZBhypro5	QuickZyme biosciences	Leiden, Netherlands
Type I collagenase	Enzyme from Clostridium histolyticum	Cat #: AAJ62406MC	Thermo Scientific	Rockford, IL
RNAzol RT	RNA preparation	Cat #: RN 190	Molecular Research Ctr	Cincinnati, OH
SYBR Green	Master Mix for rtPCR	Cat #: A25777	Applied Biosystems	Beverly, MA
High-capacity cDNA prep	RT kit	Cat #: 43-688-14	Applied Biosystems	Beverly, MA
Endotoxin	LAL Chromogenic Quant Kit	Cat #: A39552	Thermo Scientific	Rockford, IL
Lewis Lung Ca cells	LL2-DS-Red labeled	Cat #: ATCC CRL-1642	Gift, Dr. Palumbo, CCHMC	Cincinnati, OH
Steritalc	Graded mineral particle	NDC-62327-444-44	Boston Medical	Boston, MA
Talc	Ungraded mineral particle	Cat #: T2-500	ThermoFisher	Fairlawn, N.J.
Hydroxyapatite	Particle nanoXIM•HAp202	CAS No.1306-06-5	Fluidinova	Portugal
Hydroxyapatite	Spherical particles	CER-0007	Matexcel	Shirley, N.J.

Supplemental Table 4.

Gene	Primer	
	Forward	Reverse
Acp5	5'-GCCACAGTTATGTTTGTACGTG-3	5'-ACAGATTGCATACTCTAAGATCTCC-3′
Acta2	5′-CTGTTATAGGTGGTTTCGTGG A-3′	5′-GAGCTACGAACTGCCTGAC-3′
Actb	5′-ACCTTCTACAATGAGCTGCG-3′	5′-CTGGATGGCTACGTACATGG-3′
Atp6v0d2	5'-GCCAAATGAGTTCAGAGTGATG-3'	5'-AGTCTTACCTTGAGGCATTCTAC-3'
Col1α1	5'-CATTGTGTATGCAGCTGACTTC-3'	5'-CGCAAAGAGTCTACATGTCTAGG-3'
Col3α	5'-TCTCTAGACTCATAGGACTGACC-3'	5'-TTCTTCTCACCCTTCTTCATCC-3'
Csf1	5'-GGAAGATGGTAGGAGAGGGTA-3'	3'-AGGATGAGGACAGACAGGT-5'
Csf1r	5′-AGGTGTAGCTATTGCCTTCG-3′	5′-TGTATGTCTGTCATGTCTCTGC-3′
Ctsk	5'-ATCTCTCTGTACCCTCTGCAT-3′	5'-GACTCTGAAGATGCTTACCCA-3′
Fn1	5'-TTGTTCGTAGACACTGGAGAC-3'	5'-GAGCTATCCATTTCACCTTCAGA-3'
Itgb3	5'-ACAGTCATCCTCGTTCTTGTAG -3′	5'-GAACGCTCCATGAAGAAAACAC-3′
Mmp9	5'-GTGGGAGGTATAGTGGGACA-3′	5'-GACATAGACGGCATCCAGTATC-3′
Tgfb1	5'-CCGAATGTCTGACGTATTGAAGA-3'	5'-GCGGACTACTATGCTAAAGAGG-3'
Tnfsf11	5'-AGTGCTGTCTTCTGATATTCTGT -3′	5′-TCCCGCTCCATGTTCCT-3′
Tnfrsf11a	5'-CACTGTCGGAGGTAGGAGT-3'	5'-CAGGAGAGGCATTATGAGCAT-3'
Tnfrsf11b	5'-ATGCAACACATGACAACGTG-3′	5′-TGGTATAATCTTGGTAGGAACAGC-3′

Supplemental Table 4. Primers for quantitative RT-qPCR

Acp5, acid phosphatase 5; Acta2, actin α-2 smooth muscle; Actb, β-actin; Atp6v0d2, ATPase H^+ transporting v0 subunit d2; Col1α1, collagen 1α-1; Col3α1, collagen 3α-1; Csf1, colony stimulating factor 1; Csf1r, Csf1 receptor; Ctsk, cathepsin K; Fn1, fibronectin 1; Itgb3, Integrin β3; Mmp9, matrix metallopeptidase 9; Tgfb1, transforming growth factor-β1; Tnfsf11, tumor necrosis factor receptor superfamily member 11; Tnfrsf11a , tumor necrosis factor receptor superfamily member 11a; Tnfrsf11b, tumor necrosis factor receptor superfamily member 11B.
